# Pedestrian Trust in Automated Vehicles: Role of Traffic Signal and AV Driving Behavior

**DOI:** 10.3389/frobt.2019.00117

**Published:** 2019-11-28

**Authors:** Suresh Kumaar Jayaraman, Chandler Creech, Dawn M. Tilbury, X. Jessie Yang, Anuj K. Pradhan, Katherine M. Tsui, Lionel P. Robert

**Affiliations:** ^1^Department of Mechanical Engineering, College of Engineering, University of Michigan, Ann Arbor, MI, United States; ^2^Department of Electrical Engineering and Computer Science, College of Engineering, University of Michigan, Ann Arbor, MI, United States; ^3^Department of Industrial and Operations Engineering, College of Engineering, University of Michigan, Ann Arbor, MI, United States; ^4^Department of Mechanical and Industrial Engineering, University of Massachusetts, Amherst, MA, United States; ^5^Robotics User Experience and Industrial Design, Toyota Research Institute, Cambridge, MA, United States; ^6^School of Information, University of Michigan, Ann Arbor, MI, United States

**Keywords:** automated vehicles, human–automation interaction, trust in automation, virtual reality, implicit communication

## Abstract

Pedestrians' acceptance of automated vehicles (AVs) depends on their trust in the AVs. We developed a model of pedestrians' trust in AVs based on AV driving behavior and traffic signal presence. To empirically verify this model, we conducted a human–subject study with 30 participants in a virtual reality environment. The study manipulated two factors: AV driving behavior (defensive, normal, and aggressive) and the crosswalk type (signalized and unsignalized crossing). Results indicate that pedestrians' trust in AVs was influenced by AV driving behavior as well as the presence of a signal light. In addition, the impact of the AV's driving behavior on trust in the AV depended on the presence of a signal light. There were also strong correlations between trust in AVs and certain observable trusting behaviors such as pedestrian gaze at certain areas/objects, pedestrian distance to collision, and pedestrian jaywalking time. We also present implications for design and future research.

## Introduction

Automated vehicles (AVs) that can drive without the supervision or intervention of a human driver, i.e., SAE levels 4–5 (SAE-International, 2016) are becoming a reality. AVs have the potential to reduce fossil fuel consumption, improve road safety, and provide greater access to transportation (Litman, 2017). Yet, all these potential benefits depend largely on widespread public acceptance of AVs. Despite the potential benefits of AVs, public skepticism over safety is still a major barrier to AV acceptance (Liu et al., 2018; Xu et al., 2018; Zhang et al., 2019). This issue was also recently witnessed in incidents where people harassed Waymo's AVs because they felt uncomfortable and unsafe around AVs^1^. These incidents and others demonstrate both the challenges and importance of public acceptance of AVs.

To address this issue, scholars studying human–AV interactions have begun to examine the topic of human trust in AVs (Verberne et al., 2012; Choi and Ji, 2015; Basu and Singhal, 2016; Ekman et al., 2016; Du et al., 2019). However, much of this research has been directed at human–AV interactions within the AV itself. This existing research focuses primarily on identifying and examining the factors promoting drivers' trust in AVs and the implications of drivers trusting AVs (Gold et al., 2015; Verberne et al., 2015; Petersen et al., 2018; Zhang et al., 2018; Du et al., 2019). Much less attention has been paid to pedestrians and other road users outside of the AV (Saleh et al., 2017). Nonetheless, AVs have to be accepted by those who choose to ride in them as well as by pedestrians and other road users outside the AVs. Owing to the vulnerability of pedestrians in roadway interactions, there is now a growing interest in studying pedestrian trust in AVs (Saleh et al., 2017; Jayaraman et al., 2018).

Prior research on pedestrians' interaction with human-driven vehicles (HDVs) has highlighted the importance of non-verbal communication to ensure safety (Sucha et al., 2017; Rasouli and Tsotsos, 2018). Human drivers engage in non-verbal communication via eye contact, facial expressions, and hand gestures (Guéguen et al., 2015; Rasouli et al., 2018). This is often done to communicate the drivers' intent when negotiating the right-of-way with pedestrians (Sucha et al., 2017). In the absence of a human driver, it is not surprising that pedestrians have expressed concerns over not knowing or understanding the AV's intention (Merat et al., 2018; Reig et al., 2018). A clear understanding of the AV's intention is thus expected to foster trust in the AV and ultimately AV acceptance (Saleh et al., 2017; Liu et al., 2018).

AVs can communicate their intent through explicit or implicit means. Traditional methods of explicit communication in HDVs include indicator lamps, brake lamps, and horns. Current research on AV explicit communication primarily explores the efficacy of additional specialized interfaces such as light-emitting diode message boards, light-emitting diode lights, interactive head lamps, etc., in conveying vehicle intent in the absence of human drivers (Chang et al., 2017, 2018; Habibovic et al., 2018; Mahadevan et al., 2018). Although these approaches are valuable and insightful, there is currently no one standard communication interface. Moreover, when the number of AVs on the street increases in the future, explicit communication may pose problems to other road users such as information overload.

Implicit rather than explicit communication is a less explored approach to tackling the communication challenge and promoting trust between pedestrians and AVs (Dey and Terken, 2017). Implicit vehicle communication refers to the behavior cues derived from the vehicle's driving (Ackermann et al., 2018; Fuest et al., 2018). Pedestrians can get information about the AV's intent through its driving behavior, specifically through its motion and kinematics (Pillai, 2017; Ackermann et al., 2018). For example, an AV intending to yield the right-of-way to pedestrians at the crosswalk will do so by starting to slow down, whereas an AV that does not intend to yield will not slow down or may even accelerate. In this paper too, we use AV driving behavior to operationalize AV implicit communication.

The intentions of AVs can also be understood from other contextual elements such as traffic signals. AVs are expected to be much more law-abiding than human drivers (Millard-Ball, 2016; Meeder et al., 2017). Thus, under situations where the right-of-way is clear, such as at signalized crosswalks, AVs are expected to always follow the traffic rules and stop at the red light. This law-abiding nature of AVs should help foster pedestrians' trust in the AVs. Conversely, in situations where the right-of-way is unclear, pedestrians would be skeptical of AVs. One such situation is an unsignalized crosswalk, where the right-of-way varies from state to state in the United States (Shinkle, 2016). In the case of signalized crosswalks, the traffic signal clarifies the right-of-way to all traffic participants. As the AVs are always expected to follow traffic rules, the traffic signal indirectly informs the AVs' intent to the pedestrians. Traffic signals are a part of the infrastructure and dictate the right of way. Thus, they can be considered as a higher form of authority, and AVs could be expected to follow the signal irrespective of their driving behavior. Thus, the presence of a traffic signal might moderate the effects of vehicle's driving behavior on pedestrian trust. This interaction between AV driving behavior and traffic signal, is relatively less explored.

Existing research on pedestrian interaction with AVs or HDVs has predominantly focused on understanding pedestrian trust in vehicles from their behaviors such as willingness to cross, crossing speed, looking behavior while crossing, etc. (Rothenbücher et al., 2016; Rasouli et al., 2017) and use them as proxies for trust. Trust is an attitude, which is related to but different from these behaviors, which are actions. Behaviors can be moderated by environmental, cognitive, and situational factors (Lee and See, 2004). Thus, each of these different behaviors can have a different relationship with trust. These relationships between pedestrian trust and their behaviors are relatively less studied.

This paper has two major contributions. First, it contributes to the literature on pedestrian–AV trust by examining the interaction effects of AV implicit communication and crosswalk type on pedestrian trust in AVs. We consider implicit communication in the form of the AV's driving behavior: aggressive, normal, and defensive. Second, we examine the effects on both self-reported trust and trusting behavior and examine the relationships between trust and trusting behaviors. This study goes beyond other studies of pedestrian–AV interaction by examining the impacts of the situation in which the pedestrian–AV interaction takes place: unsignalized and signalized (with traffic signals) crosswalks. To accomplish this, we conducted a user study to investigate the moderation effects of traffic signal on impact of AV driving behavior on participants' self-reported trust (i.e., attitude) and their trusting behavior (i.e., actions). By measuring both self-reported trust and trusting behaviors, we were able to provide greater clarity with regards to their relationship in the study of pedestrian–AV interactions. Overall, the results provide new insights into trust between pedestrians and AVs.

The rest of the paper is organized as follows. Section Background and Related Work presents the background and existing research on impacts of AV driving behavior and traffic signal on pedestrians' behavior and trust in AVs. Sections Research Model and Method describe the proposed research model and experimental methodology, respectively. Section Results reports the results of a virtual reality user study. Sections Discussion and Limitations discuss the implications and limitations of the research, respectively.

## Background and Related Work

Although pedestrian interaction with HDVs has been studied extensively (Schmidt and Färber, 2009; Schneemann and Gohl, 2016; Rasouli and Tsotsos, 2018), only recently have scholars started to explore pedestrian interactions with AVs (Pillai, 2017; Deb et al., 2018; Fuest et al., 2018; Jayaraman et al., 2018).

Research on implicit communications between pedestrians and AVs has focused on the problems with the absence of the human driver. The AV's driving behavior has been used as a form of implicit communication. Typically, researchers have varied AV driving behavior from more to less aggressive by varying the vehicle's velocity profile and measured participants' responses to the driving behavior (Pillai, 2017; Schmidt et al., 2019). Studies have shown that AVs can implicitly communicate their intent to pedestrians through their driving behavior (Fuest et al., 2018; Schmidt et al., 2019). For example, Fuest et al. (2018) examined AV intent recognition by pedestrians. They used a “Wizard of Oz” (WOZ) approach where the driver wore a car seat costume and hid in plain sight from the pedestrians. Results indicate that pedestrians in general were able to identify the AV's intent of stopping or not stopping from its driving behavior.

Scholars have also begun examining the impact of AV driving behavior on pedestrian's trust. Pedestrian trust in AVs is highly relevant because pedestrians were more wary of crossing in front of an AV without a driver than crossing in front of a HDV (Lagstrom and Lundgren, 2015), indicating less trust in AVs. In this paper, we use the definition of trust from Lee and See (2004) that defines trust as “the attitude that an agent will help achieve an individual's goals in a situation characterized by uncertainty and vulnerability.” In our study, trust is the attitude of the pedestrians that the AV would help them in their goal to cross the street.

Existing studies varied the AV driving behaviors and explored pedestrian trust through behaviors such as willingness to cross, crossing paths, and comfort of crossing (Rothenbücher et al., 2016; Pillai, 2017; Zimmermann and Wettach, 2017). For example, Rothenbücher et al. (2016) explored the reactions of pedestrians upon encountering an AV. They found that people generally crossed the street normally and were tolerant of aggressive driving by the AV. Pedestrians' willingness to cross seemed to be unaffected by the AV's different driving behaviors. However, both Pillai (2017) and Zimmermann and Wettach (2017) found that when the AV engaged in what would be considered more defensive driving behavior (decelerating early), vs. more aggressive driving behavior (decelerating later), they perceived the defensive AV to be more controlled and reliable than the aggressive AV. Overall, there is more evidence that different AV driving behaviors can affect pedestrian trust differently.

To the authors' knowledge, there are currently no studies examining the role of the traffic signal on pedestrian's trust in AVs. However, several existing studies on pedestrian–HDV interactions have shown that pedestrians express more trusting behavior around signalized crosswalks. For example, Asaithambi et al. (2016) found that pedestrians accepted shorter time gaps and crossed closer to the vehicles after a traffic signal was installed at an intersection. They also observed other trusting behaviors such as reduced walking speed and increased waiting time after the installation of the traffic signal, indicating more trusting behaviors at signalized crosswalks. This finding agrees with Tom and Granie (2011), who found that pedestrians were more cautious of the oncoming vehicles and looked at vehicles more during the unsignalized crosswalks than signalized crosswalks before crossing the street. Similarly, Rasouli et al. (2017) evaluated pedestrian communication behavior and found that pedestrians are more cautious and less likely to cross the street after communicating their intention (by looking at the oncoming vehicles) if the crosswalk is not signalized and more likely to cross if some form of signal is present. Overall, signalized crosswalks are generally considered safer by pedestrians as they clarify the right of-way, and thus, it can be expected that pedestrians would exhibit more trusting behaviors around signalized crosswalks. Nonetheless, we should acknowledge at least one study whose findings contradict these results. Hatfield and Murphy (2007) found that pedestrians exhibited more trusting behavior at unsignalized crosswalks, in that they were more distracted and did not pay attention to the traffic and the street while crossing at unsignalized crosswalks than signalized crosswalks.

Although there is a fair understanding of the individual effects of vehicle driving behavior and traffic signal on pedestrian behavior, the interaction effect of the two is not clearly understood. This is important especially in the case of pedestrian–AV interactions as the absence of a human driver may place more reliance on the traffic signal to understand the AV intentions.

The relationship between trust as an attitude and the observable trusting behaviors during pedestrian–vehicle interactions is relatively unknown. Existing research has focused on understanding pedestrian trust through their behaviors, which we refer as trusting behaviors (Rothenbücher et al., 2016; Pillai, 2017; Rasouli et al., 2017; Zimmermann and Wettach, 2017). Trust is related to but different from trusting behaviors. Trust is an attitude, whereas trusting behaviors are actions. Azjen (1980) developed a framework to clarify these differences, which shows that behaviors result from intentions and intentions are a function of attitudes. Trust in automation studies (Lee and Moray, 1994; Riley, 1996; Lee and See, 2004) have identified various factors affecting trusting behavior. Generally, trust is only one of the factors that influence behaviors, in addition to workload, situational awareness, system capability, and other contextual and environmental factors (Lee and See, 2004). Although trust is related to trusting behaviors, the relationship between trust and trusting behaviors might not be straightforward.

Thus, existing research on pedestrian–AV interactions has not clearly established the moderation effect of traffic signal on the impact of AV driving behavior on pedestrian trust and behavior. Furthermore, the relationships between pedestrian's subjective trust and their observable behaviors while crossing a street is not well-known. In this study, we address both of these shortcomings by conducting a human–subjects study under different conditions of AV driving behavior with the presence/absence of a traffic signal and measuring pedestrians' trust and behaviors.

## Research Model

### Uncertainty and Pedestrians' Trust in AVs

Uncertainty is defined as the inability to predict another's behavior because of a lack of information about the person or environment (Baxter and Montgomery, 1996; Kramer, 1999). When individuals meet, they communicate and exchange information as a means of reducing uncertainty with regards to each other's intentions. The more information gained, the less uncertainty one has about the other individual or situation. However, when direct communication with an individual is not possible, people seek information from third parties or through observation (Sunnafrank, 1986).

As uncertainty increases, humans are more motivated to engage in information seeking to reduce uncertainty. Furthermore, uncertainty decreases as the amount of information communicated increases. In other words, the more the uncertainty, the more the people seek information to reduce it; the more information provided, the less uncertainty. Trust and uncertainty are inversely related (Lewis and Weigert, 1985; Colquitt et al., 2012). The greater uncertainty one has about the outcome of an interaction with an agent, the less trust one has in that agent (Robert et al., 2009; Colquitt et al., 2012). Likewise, the more trust someone has in an agent, the less the uncertainty regarding the outcome of an interaction with that agent. Thus, availability of information plays an integral role in improving trust by reducing uncertainty. For example, Helldin et al. (2013) found that when the AV informed the uncertainty in its ability to drive to the human driver, the trust in the AV increased. In this paper, we consider the information about AV's intent to be available from the AV's driving behavior and the traffic signal. Unlike Helldin et al. (2013), we do not quantify uncertainty but use the qualitative relationship between trust and uncertainty from AV driving behavior and/or absence of traffic signal to develop our hypotheses.

### Hypotheses

In our research, we used uncertainty and availability of information to understand a pedestrian's trust in AVs. When a pedestrian approaches a crosswalk, there is some degree of uncertainty about an AV's actions—Will the AV stop? If so, will it stop within a safe distance, and will it remain stopped to allow the pedestrian to cross safely? Pedestrians would attempt to reduce this uncertainty by seeking information to help them predict the AV's actions. In our study, the information about the AV's actions can be directly estimated from the AV's driving behavior and/or can be obtained from the traffic signals which determine the right of way for all traffic participants. The more information available to facilitate the pedestrian's prediction of the AV's actions, the less uncertainty and the more trust they should have in the AV. Conversely, the less information available, the more uncertainty and the less trust they should have in the AV.

Thus, this paper's premise is:

*As the available information that allows the pedestrian to predict the actions of the AV increases, so should trust in the AV*.

Our study included two sources of information: the AV's driving behavior and the traffic signal. Driving behavior is typically classified into defensive, normal, and aggressive behaviors (Mizell et al., 1997; Steimetz, 2008; Schneemann and Gohl, 2016). Defensive driving is characterized as slow and predictable, normal driving less so, while aggressive driving is characterized by unpredictable behavior including high speeds, delayed stopping, or not yielding the right-of-way (Mizell et al., 1997; Steimetz, 2008). For example, a defensively driving AV might sense a pedestrian trying to cross the street and could start to slow down very early to indicate its intent of yielding to the pedestrian, even though legally it may have been the AV's right-of-way. On the other hand, an AV driving more aggressively might slow down and yield late or might even accelerate to indicate that it is not yielding to the pedestrian. Thus, in scenarios where a pedestrian walks onto the road, an aggressive AV would brake later and harder than a defensive AV to avoid a potential collision with the pedestrian (Zimmermann and Wettach, 2017). This makes it hard to predict whether an aggressive AV would ever slow down or stop for a pedestrian. The unpredictability of aggressive driving should lead to low trust in AVs. Thus, the more aggressive the vehicle drives, the more uncertain its behavior, and the lower the trust in AVs.

Pedestrians can also gather information from the surroundings—road type, location of stop sign, traffic signal, etc. Vehicles are expected to stop at traffic signals. Thus, the state of the signal would provide information about what the vehicles are expected to do. Particularly, AVs are expected to be more law abiding, and thus, their intent would be more predictable. Therefore, signalized crosswalks should decrease uncertainty and increase AV trust by clarifying who should stop, whereas at unsignalized crosswalks, the right of way is less clear (Shinkle, 2016).

**Figure 1:**
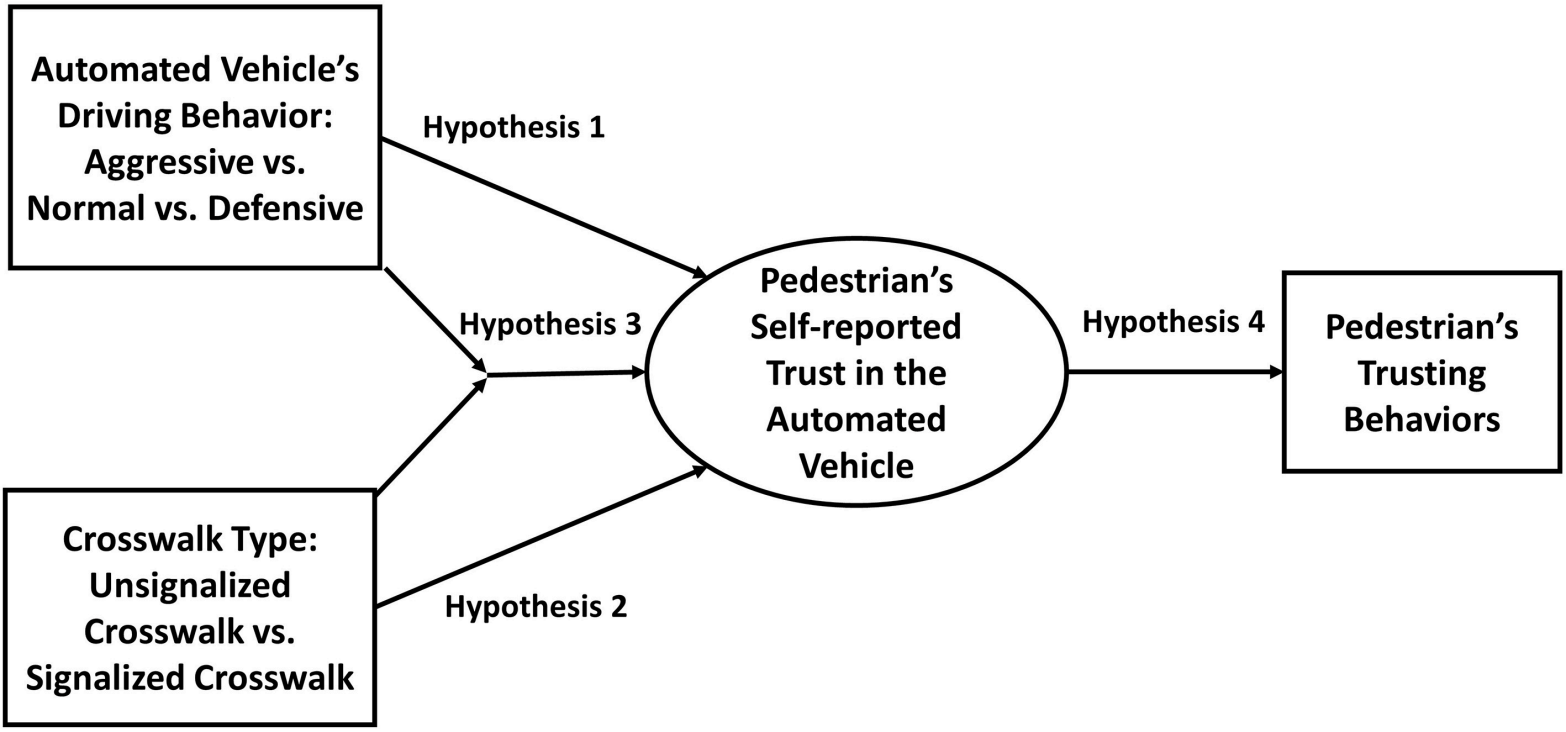
Pedestrian–automated vehicle (AV) trust model.

Furthermore, the crosswalk type should moderate the impacts of aggressive driving. The presence of a traffic signal should reduce the negative impact of aggressive driving on AV trust. Individuals should be more likely to believe that the AV will stop regardless of its driving behavior. Therefore, aggressive driving should have a weaker impact on AV trust at signalized crosswalks. Finally, although the relation between trust and trusting behavior may not be straightforward, we expect increased trust in the AVs to generally result in more trusting behaviors. Simply put, the more an individual trusts the AV, the more he or she should engage in trusting behaviors with regard to the AV. Our research model is graphically summarized in Figure 1.

We test the following hypotheses:

H1: Aggressive AV driving behavior decreases pedestrians' trust in AVs.
H2: Signalized crosswalk increases pedestrians' trust in AVs.
H3: Crosswalk type moderates impact of aggressive AV driving behavior.
H4: Pedestrians' trust in AVs engenders more trusting behaviors from pedestrians.

## Methods

### Study Participants

We recruited participants through email and obtained informed consent from each participant. Thirty participants, of which 28 were college students, joined in this study (9 female), with a mean age of 22.5 years [standard deviation (SD) = 2.8 years]. The study population was relatively young as it appealed primarily to the student population in the university, and we did not explicitly control for age during subject recruitment.

### Development of Experimental Apparatus

**Figure 2:**
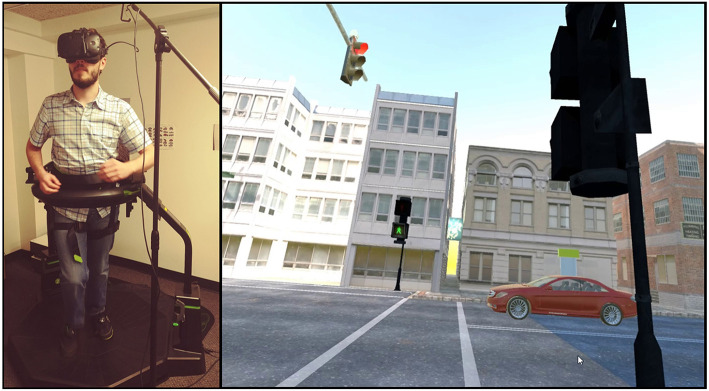
Virtual reality setup for user study. The left side shows the user wearing the HTC Vive headset and walking on the omni-directional treadmill. The right side shows the virtual environment as seen by the participant.

Participants were placed in an immersive virtual environment (IVE) with an HTC Vive virtual reality headset (Vive; HTC Corp., New Taipei, Taiwan), walking on an omni-directional treadmill (Virtuix Omni; Virtuix Inc., Austin, TX); they took on the role of a pedestrian walking in an urban environment. The left side of Figure 2 shows the equipment setup, while the right side shows the scene from participants' point of view as they wore the headset and walked on the treadmill. We developed the urban scenario simulation to be as realistic as possible. During the experiment, participants crossed a street at a mid-block crosswalk with several oncoming AVs. The street was one way with two lanes for the AVs. The AVs in this study were fully automated without any humans inside and produced engine sounds based on speed of the AV and distance of the AV to the pedestrian. We manipulated the type of driving behavior (defensive, normal, and aggressive) and the type of crosswalk (signalized and unsignalized). We employed a within-subjects experimental design so every participant experienced all six conditions (3 × 2). Sample videos of the six treatment conditions are available online^2^ for reference. The IVE was built using the Unity Game Engine (Unity Technologies, San Francisco, CA). The treadmill senses feet movements and torso orientation to provide a direction and speed for movement in the virtual environment that matches the participant's input in the physical world.

### Experimental Task

**Figure 3:**
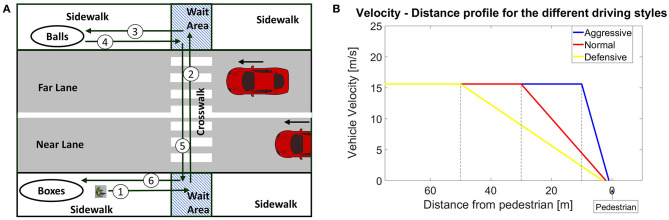
**(A)** Pedestrian state divisions in the virtual environment. Numbered arrows indicate a typical pedestrian path while doing the task. **(B)** Driving profiles for the three driving behaviors when the pedestrian is on the road in the same lane as the automated vehicle (AV). To achieve the specified stopping distance and slow speeds, defensive behavior decelerated much earlier than normal or aggressive.

In the experiment, participants were asked to move three numbered balls, one at a time, from one side of the street to the other, placing them in corresponding numbered boxes. Participants were required to remember the ball's number, which disappeared after it was picked up. The ball task was designed such that the crossing activity was embedded within the overall task of moving the balls. This served two purposes. First, it allowed participants to make multiple street crossings without experimenters explicitly instructing them to make such crossings. Second, the task was designed to reduce any participant reactivity, such as from an observer effect, wherein participants' actual crossing behaviors could be affected by their knowledge that the experimenters were specifically measuring such behaviors (Baum et al., 1979). This ball task helped disguise from the participants the fact that their crossing behaviors were of primary interest to the experimenters. The activities for moving a ball include approaching the crosswalk, waiting to cross, crossing the street, approaching the ball, picking the up the ball, approaching the crosswalk, waiting to cross, crossing the street, approaching the boxes, and dropping the ball. The numbers in Figure 3A describe a typical sequence of pedestrian movements. Thus, by performing the ball-task, they had to cross the street six times.

### Design of Interaction Scenarios

The interaction scenarios were designed to mimic a downtown urban crosswalk. Figure 3A shows the layout of the virtual reality environment. Participants could move around all the different areas including the sidewalks, the road lanes, and the wait areas. The wait areas are where the pedestrian would typically wait before crossing the road. Participants encountered AVs while crossing in either direction.

In both signalized and unsignalized conditions, all AVs approached the crosswalk at a constant speed of 15.6 m/s (35 mph). For each treatment condition, the vehicle changed its driving behavior when it encountered a pedestrian within its reaction distance (refer to Table 1). This distance signifies the attentiveness of the different driving behaviors. As discussed earlier, the unpredictability of aggressive driving can be attributed to the delayed stopping or failing to yield the right of way (Mizell et al., 1997) by the AV. We defined the aggressive driving behavior to be less cautious, by which we made AVs with aggressive driving behavior react to pedestrians much later than the defensive or normal driving conditions. We defined this behavior by varying the reaction distance, which is the distance from the pedestrian the AV would start reacting to pedestrians. We also varied the AVs' reactions to the pedestrian such as stopping, slowing down, or not slowing based on the position of the AVs and the pedestrian. The different driving behaviors were obtained by tuning the AVs' reactions, reaction distance, and driving parameters such as acceleration and speed across the three behaviors. The resulting behaviors were perceived to be different from one another during internal validation. The change in vehicle behavior is based on the discrete location of the pedestrian as described in Table 1. The stopping distance in Table 2 refers to the distance between the pedestrian and the vehicle when it is stopped.

**Table 1:** Different vehicle reactions to various pedestrian positions characterizing the different driving behaviors.

**Behavior**	**Pedestrian position**				**Reaction distance (m)**
	**Sidewalk**	**Wait area**	**Same lane as vehicle**	**Other lane as vehicle**	
Defensive	Full speed	Slow speed	Stop	Stop	50
Normal	Full speed	Slow speed	Stop	Slow speed	30
Aggressive	Full speed	Full speed	Stop	Full speed	10

**Table 2:** Vehicle parameters characterizing the different driving behaviors.

**Behavior**	**Stopped distance (m)**	**Maximum acceleration (m/s^**2**^)**	**Slow speed (m/s)**	**Full speed (m/s)**
Defensive	3	3	4	15.6
Normal	2	5	7	15.6
Aggressive	1	8	NA	15.6

The cars always stop before the crosswalk if there is a pedestrian on the street. The cars do not stop when pedestrians are waiting/walking on the sidewalk. However, to elicit realistic pedestrian behavior, participants were not explicitly told that AVs would always stop if they are on the road. Furthermore, the AVs with the same driving behavior do not react in the exact same way each time as their deceleration rates depend on the relative position between the pedestrian and AVs when the pedestrian reach the particular positional states.

Table 1 provides the discrete AV driving behavior model based on the pedestrian's positional state, and Table 2 provides the vehicle parameters used in the study. Typical driving profiles for the three driving behaviors are shown in Figure 3B. For a pedestrian in the wait area, during the normal driving behavior conditions, the vehicles in the near lane and far lane slowed down from 15.6 to 7 m/s. They started slowing down at a distance of 30 m from the pedestrian. When the pedestrian stepped into the near lane, the vehicle in near lane stopped 2 m from the pedestrian, whereas the vehicle in far lane continued at a speed of 7 m/s.

In addition, in the signalized conditions, the AV stopped at the appropriate stopping distances (Table 2) when the signal was red or yellow and maintained the same behavior as in unsignalized conditions (Table 1) when the signal was green. In signalized conditions, when the pedestrian was not on the road, the stopping distance refers to the distance between the vehicle when it was stopped and the center of the crosswalk. The behaviors across signalized and unsignalized conditions were maintained to be as similar as possible for experimental validity and to avoid any confounding effects due to variations in the vehicle behaviors, when examining the effect of traffic signal.

The signal for the vehicles operated on a 38 s cycle: green for 20 s, yellow for 3 s, then red for 15 s (Urbanik et al., 2015). The cycle ran continuously on the background, but the signal changed to yellow and red only after the participant pressed the provided signal button. If the signal button was not pressed by the participant, the signal remained green. Vehicular traffic was generated in a predetermined pseudo-random sequence of short (3 s) and long (5 s) gaps. The probability of a short gap occurring was 75%, and a long gap was 25%, inducing the participants to observe the cars during the short gaps while waiting for a long gap to occur to cross. Vehicles were generated in both lanes of the street, going in the same direction.

### Training

Participants underwent two training sessions before the start of testing. In the first training session, there were no vehicles on the road and participants practiced the task of picking the balls from one side of the road and placing them on a receptacle on the other side, until they were comfortable doing the task. In the second training session, participants were fenced so that they could not be able to enter the road but see the AVs on the road. In this session, AVs were shown to pedestrians so that they can see how the AVs looked like and not be surprised when they see them during the actual scenarios. AVs with a constant speed of 35 mph (15.6 m/s) were traveling on the road but did not react to the pedestrians. However, the behaviors of the AVs during the actual treatment conditions followed the behaviors defined by the parameters in Tables 1, 2.

We employed a within-subjects experimental design. After the training sessions described above, participants experienced each of the six treatment conditions (defensive, normal, and aggressive driving behaviors for each of signalized and unsignalized crosswalks) once. The conditions' sequence was counterbalanced using a Latin square design (Lewis, 1989). The standard Latin square design that we employed in the study is available in Appendix 1.

The balanced Latin square design has a group of sequences of treatment conditions such that every condition appears before and after every other condition exactly once. This design helps to compensate for immediate sequential effects (Lewis, 1989).

### Measurements

We collected attitudinal, behavioral, physiological, and other self-reported measures. We measured participants' propensity to trust (Preusse and Rogers, 2016) and experience with virtual reality before the experiment (calculated as a mean of 1–7 Likert scale responses). After each treatment condition, participants gave 1–7 Likert scale ratings measuring trust and perceived AV aggressiveness. For measuring self-reported trust, we adapted the Muir scale questions (Muir, 1987), a highly validated trust in automation scale. We modified the questions to reflect the pedestrian–AV interaction context (refer Appendix 2). Self-reported trust was calculated as the mean of the responses to the trust questionnaire. We also measured simulator sickness, calculated using the items and procedure mentioned in Kennedy et al. (1993) (refer Appendix 3), at the end of the experiment.

We collected six dependent measures of trusting behavior from the simulation, some of which were calculated for each of the six crossings within a treatment condition and averaged. *Average distance to collision* measured how close a participant was to being hit by the AV as the distance between the AV in its lane and the participant when he/she entered that lane. *Average jaywalking time* was the average time participants spent either crossing the street when the AV had the right of way, which was whenever the pedestrian signal was red in the unsignalized conditions, or crossed the street outside of the crosswalk in both the signalized and unsignalized conditions. *Average wait time* measured the average time they spent waiting before they crossed the street. *Average crossing speed* measured how fast they crossed the street. *Average crossing time* was the average duration of the crossing. *Overall task time* measured how long they took to complete the entire treatment condition.

We examined participants' eye gaze to explore its relationship with self-reported AV trust. A lack of monitoring is related to high trust in automation (Hergeth et al., 2016). We divided the environment into seven areas of interest (AOI): (1) looking at approaching AVs, (2) checking for AVs, (3) pedestrian signal light, (4) traffic light, (5) task materials, (6) crosswalk and buildings directly across the crosswalk, and (7) everything else in the environment that included the sky, other buildings along the road, and roads not in the crosswalk region. The crosswalk and buildings directly across represented regions when a participant stared ahead. We measured the duration of time each participant spent looking at the different AOI using the Pupil Labs eye tracker (Pupil; Pupil Labs, Berlin, Germany).

Other research studies have performed *post-hoc* frame-by-frame manual coding to identify the AOI (Tapiro et al., 2014; Trefzger et al., 2018). In our study, the AOI at which the participants gazed was identified in real time by interfacing the Pupil Labs eye tracker with the Unity simulation. At every sampling instant, the Unity simulation obtained the gaze point from the eye tracker and cast a ray to automatically identify which AOI intersected with the gaze ray.

## Results

The descriptives (mean and standard deviation) of our survey measurements are reported in **Table 4**. Having a within-subjects experimental design, our study collected repeated measurements (for each of the six treatment conditions) from the same subject. To account for this non-independence in the data, we employed mixed linear repeated modeling (MLRM) technique (Stroup, 2012) to understand the relationships between the dependent and independent variables. MLRM makes it easy to study the effects of covariates in addition to the treatment variables on the dependent variable. We used SPSS v24 (IBM, Armonk, NY) for all our analyses. The data used for the analyses and the SPSS analyses codes are available as Supplementary Materials.

### Manipulation Check of Aggressive Driving

**Figure 4:**
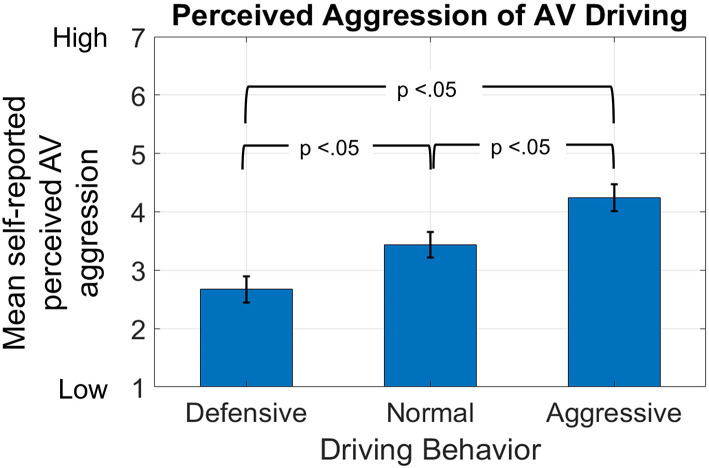
Manipulation check of aggressive driving. Perceived aggression of automated vehicle (AV) driving is lowest for defensive driving and highest for aggressive driving conditions.

We conducted a manipulation check to verify if the participants perceived each of the driving conditions to have different levels of driving aggression. To accomplish this, we ran a MLRM with driving condition as the independent variable and the perceived AV driving aggression as the dependent variable. The model revealed a significant difference (*p* < 0.001) among the driving conditions. The mean (standard deviation) values were *x* = 2.67 (0.22) for defensive, *x* = 3.44 (0.21) for normal, and *x* = 4.24 (0.23) for aggressive driving conditions. As shown in Figure 4, all pairwise comparisons were significantly different from one another (*p* < 0.05). Our results indicate that our manipulation of driving behavior was successful.

### Measurement Validity

To verify if our survey constructs measured what they were intended to measure, we conducted a factor analysis to examine convergent and discriminant validity of the self-reported trust and simulator sickness measures (see Table 3). Only one item (Trust: Reliability) did not meet the 0.7 loading requirement indicating convergence validity. In addition, no crossloadings exceeded 0.3, indicating discriminant validity. Thus, the results indicate both discriminant and convergent validity (Fornell and Larcker, 1981).

**Table 3:** Survey measurement validity using factor and cross loadings of trust and simulator sickness survey measures.

**Item**	**Self-reported AV trust**	**Simulator sickness (SS)**
Trust: competence	**0.83**	0.12
Trust: predictability	**0.86**	0.01
Trust: dependability	**0.86**	0.09
Trust: responsibility	**0.84**	0.05
Trust: reliability	**0.62**	0.02
Trust: faith	**0.72**	0.00
SS: disorientation	0.07	**0.91**
SS: nausea	0.06	**0.82**
SS: oculomotor	0.01	**0.91**

*Convergent validity: factor loadings > 0.7; Discriminant validity: cross loadings < 0.3*.

*Bold values indicate the representative factors included in the measures in each column*.

To maintain content validity and consistency with previous studies, we included the item with the low factor loading of 0.62. In addition, reliabilities of both self-reported trust (α = 0.92) and simulator sickness (α = 0.85) exceeded the 0.7 recommendation (Carmines and Zeller, 1979). Low correlations were observed among all the measured variables. This provided evidence of discriminant validity among the variables. Specifically, all correlations were below 0.5 (see Table 4).

**Table 4:** Descriptives of measurements and correlations between the measurements.

	**Parameters**	**Mean**	**SD**	**1**	**2**	**3**	**4**	**5**	**6**	**7**
1	Trust	5.68	1.10							
2	Aggressive driving	3.47	1.83	−0.47^**^						
3	Signalized crosswalks	0.50	0.50	0.33^**^	−0.31^**^					
4	Driving condition	0.50	0.50	−0.08	0.28^**^	0.00				
5	Age	22.50	2.76	0.06	−0.06	0.00	0.00			
6	Propensity to trust	5.33	0.46	0.20^**^	−0.31^**^	0.00	0.00	−0.14		
7	Virtual reality experience	3.36	1.20	0.01	−0.01	0.00	0.00	−0.34^**^	0.20^**^	
8	Simulator sickness	28.30	23	−0.10	0.31^**^	0.00	0.00	0.22^**^	−0.42^**^	−0.24^**^

** *Correlation is significant at the 0.01 level (two-tailed)*.

*Trust is calculated as the mean of the responses (1–7 likert scale) from the survey in Appendix 1*.

*Aggressive driving is a rating of perceived aggression (1–7 Likert scale) of the AV driving*.

*Signalized crosswalks is a boolean variable for presence/absence of traffic signal*.

*Driving condition is a boolean variable for low-aggressive and high-aggressive behavior*.

*Propensity to trust is calculated as the responses (1–7 likert scale) from the Complaceny rating survey (Preusse and Rogers, 2016)*.

*Virtual Reality experience is measured on a 1–7 Likert sclae before the experiment starts*.

*Simulator sickness is calculated using the formula in Kennedy et al. (1993)*.

Before conducting our analyses, we checked for heteroscedasticity by performing Glejser test, which states that variables have non-linear and unequal variances if *p* < 0.05 (Glejser, 1969). We found evidence of both non-linearity and unequal variances related to average distance to collision (*p* = 0.01), average jaywalking time (*p* = 0.03), and average crossing speed (*p* = 0.01). To improve the linearity and equality of the variances, we performed log transformations on each dependent variable and verified the absence of heteroscedasticity (*p* ≥ 0.05 for all dependent variables).

### Population Effects

We found that neither age (fixed effects estimate, β = 0.03, *p* = 0.77) nor gender (fixed effects estimate, β = −0.22, *p* = 0.40) had significant effects on the self-reported trust in AVs. The effect of age was not significant perhaps because of the limited age range of our study population. The study had a fairly young population (18–30 years) with a mean age of 22.5 years [and standard deviation (SD) = 2.8 years].

### Hypothesis Testing

For our analysis, we used the self-reported perceived AV aggression as an independent variable because it is a more accurate measure of how the pedestrians actually perceived the AVs' behavior, which in turn would affect their trust. We used the self-reported trust, calculated as the mean of the responses to the trust survey, as the dependent variable. We conducted the analysis for H1–H3 in two parts. First, we analyzed the main effects model with the control variables and the variables measuring aggressive driving (self-reported perceived AV aggression) and crosswalk type. Second, we included the moderation effect involving signalized crosswalks and aggressive driving. We employed the full model with the moderation effect because it had a lower Schwarz's Bayesian information criterion (= 472) than the model with only the main effects (Schwarz's Bayesian information criterion = 500) and thus fit the data better (Stone, 1979). The full model and correlations are shown in Tables 4, 5.

**Table 5:** Trust model: higher trust during less aggressive driving and during presence of signal with presence of signal moderating the effect of aggressive driving on trust.

**Independent parameter**	**Estimation (β)**	**SE**	***df***	***t***	**Sig**.	**95% CI**	
Intercept^*^ (γ_00_)	4.93	0.23	63.76	21.86	0.00	4.49	5.39
Aggressive driving^*^ (γ_01_)	−1.08	0.22	76.96	−4.97	0.00	−1.51	−0.65
Signal condition^*^ (γ_10_)	0.41	0.12	85.16	3.50	0.00	0.18	0.65
Aggressive driving × signal condition^*^ (γ_11_)	0.40	0.12	91.67	3.43	0.00	0.17	0.64
Driving condition (γ_20_)	0.10	0.06	49.68	1.76	0.08	−0.01	0.21
Age (γ_02_)	−0.01	0.12	21.47	−0.08	0.93	−0.27	0.25
Propensity to trust (γ_03_)	0.11	0.13	21.41	0.88	0.39	−0.15	0.38
Virtual reality experience (γ_04_)	−0.01	0.12	21.29	−0.03	0.97	−0.26	0.25
Simulator sickness (γ_05_)	0.03	0.13	22.72	0.21	0.83	−0.24	0.30
**Independent parameter**	**Estimation (*****β*****)**	**SE**	**Wald** ***Z***		**Sig**.	**95% CI**	
Random intercept variances (ν_0j_)	0.21	0.14	1.49		0.14	0.06	0.77
Random signal condition variances (ν_*1*j**_)	0.03	0.03	0.99		0.32	0.00	0.24
Random driving condition variances (ν_*2*j**_)	0.01	0.02	0.64		0.52	0.00	0.21
Residual variances (*ε_*ij*_*)							
Treatment condition 1 (Defensive Unsignalized)^*^	0.98	0.28	3.52		0.00	0.56	1.71
Treatment condition 2 (Normal Unsignalized)^*^	0.52	0.16	3.35		0.00	0.29	0.94
Treatment condition 3 (Aggressive Unsignalized)^*^	1.14	0.33	3.44		0.00	0.65	2.02
Treatment condition 4 (Defensive Signalized)^*^	0.21	0.08	2.77		0.01	0.11	0.44
Treatment condition 5 (Normal Signalized)^*^	0.31	0.09	3.38		0.00	0.01	0.44
Treatment condition 6 (Aggressive Signalized)	0.07	0.06	1.10		0.27	0.01	0.39

* *Significant model parameters*.

*Fixed effects estimates (β) indicate the direction and degree of relationship between trust and the model variables*.

We derived our mixed linear model from both level 1 (Equation 1) and level 2 (Equation 2) equations (Hoffman and Rovine, 2007). This two-level modeling accounts for the effects of both group-level and individual-level variables and allows random variations for the group-level variables (Stroup, 2012). In the level 1 equation, *Y*_*ij*_ is the trust outcome of individual *i* (from 30 subjects) in group *j* (from 6 treatment conditions). β_0*j*_ represents the group intercept values, β_1*j*_ and β_2*j*_ represent the effects of group predictors *SignalCondj* and *DriveCondj*, respectively, whereas β_01_, β_02_, β_03_, β_04_, and β_05_ represent the effects of the individual predictors *Aggress*_*ij*_*, Age*_*ij*_, *ProTrust*_*ij*_, *V irReaExp*_*ij*_, and *SimSic*_*ij*_, respectively. ε_*ij*_ represents the residual for individual *i* in group *j*.

(1) $$\begin{array}{l} Y_{ij}=\beta_{0j}+\beta_{1j}\left(SignalCond_{j}\right)+\beta_{2j}\left(DriveCond_{j}\right)\\ +\beta_{01}\left(Aggress_{ij}\right)+\beta_{02}\left(Age_{ij}\right)+\beta_{03}\left(ProTrust_{ij}\right)\\ +\beta_{04}\left(VirReaExp_{ij}\right)+\beta_{05}\left(SimSic_{ij}\right)+\varepsilon_{ij} \end{array}$$

Group level variables are associated with varying intercepts shown in the level 2 equations (Equation 2). Gammas γ_00_, γ_10_, and γ_20_ represent the intercepts (fixed main effects), while ν_0*j*_, ν_1*j*_, and ν_2*j*_ represent their corresponding variances. These variances highlight that β_0*j*_, β_1*j*_, and β_2*j*_ are allowed to randomly vary. Gammas γ_01_, γ_02_, γ_03_, γ_04_, and γ_05_ represent the intercepts (fixed main effects) for their corresponding individual-level counterparts, whereas γ_11_ represents the effect of the interaction term *Aggress*_*ij*_ * *SignalCond*_*j*_. β_01_, β_02_, β_03_, β_04_, and β_05_ are not allowed to randomly vary and therefore do not have corresponding variances.

(2) $$\begin{array}{l} \beta_{0j}=\gamma_{00}+\nu_{0j}\\ \beta_{1j}=\gamma_{10}+\gamma_{11}\left(Aggress_{ij}\right)+\nu_{1j}\\ \beta_{2j}=\gamma_{20}+\nu_{2j}\\ \beta_{01}=\gamma_{01}\\ \beta_{02}=\gamma_{02}\\ \beta_{03}=\gamma_{03}\\ \beta_{04}=\gamma_{04}\\ \beta_{05}=\gamma_{05} \end{array}$$

Our mixed linear model is derived by substituting Equation (2) into Equation (1). The final model we used is shown in Equation (3).

(3) $$\begin{array}{l} Y_{ij}=\gamma_{00}+\gamma_{01}\left(Aggress_{ij}\right)+\gamma_{02}\left(Age_{ij}\right)+\gamma_{03}\left(ProTrust_{ij}\right)\\ \;+\gamma_{04}\left(VirReaExp_{ij}\right)+\gamma_{05}\left(SimSic_{ij}\right)+\gamma_{10}\left(SignalCond_{j}\right)\\ \;+\gamma_{11}\left(Aggress_{ij}\right)*\left(SignalCond_{j}\right)\\ \;+\gamma_{20}\left(DriveCond_{j}\right)+\nu_{0j}+\nu_{1j}\left(SignalCond_{j}\right) \end{array}$$

We also tested H4 using a mixed linear modeling approach (Table 6). We used the mean of trust in the AV per condition as the independent variable when predicting trusting behaviors. To justify the aggregation by condition, we calculated the intraclass correlation coefficient (ICC). ICC measures the degree to which an individual level variable is influenced by group level membership. The higher the ICC, the more the individual-level variable is driven by group membership, and the more justification one has to create a group-level variable. The ICC value of trust in the AV per condition was 0.44, exceeding 0.10 (Bliese, 2000), indicating that a group variable was valid.

**Table 6:** Mixed linear models of trust and each trusting behavior separately with trust being the dependent variable predicting trusting behaviors.

**Parameter**	**Estimation (β)**	**SE**	***df***	***t***	**Sig**.	**95% CI**	
Intercept	0.88	0.01	75.63	87.43	0.00	0.86	0.90
CL trust	−0.38	0.01	68.60	−38.71	0.00	−0.40	−0.36
Dependent Variable: Log of average distance to collision^*^ (m)							
Intercept	−0.08	0.04	17.37	−1.79	0.09	−0.17	0.01
CL trust	0.17	0.04	13.84	3.78	0.00	0.07	0.26
Dependent Variable: Log of average jaywalking time^*^ (s)							
Intercept	16.24	0.53	92.74	30.60	0.00	15.19	17.30
CL trust	4.26	0.54	63.37	7.92	0.00	3.19	5.34
Dependent Variable: Average waiting time^*^ (s)							
Intercept	222.58	4.33	132.34	51.45	0.00	214.02	231.14
CL trust	32.31	4.24	75.75	7.63	0.00	23.88	40.76
Dependent Variable: Overall task time^*^ (s)							
Intercept	3.86	0.10	165.42	39.40	0.00	3.67	4.06
CL trust	−0.08	0.10	105.36	−0.82	0.42	−0.28	0.12
**Dependent Variable: Average crossing time (s)**							
Intercept	0.27	0.01	173.80	32.28	0.00	0.25	0.29
CL trust	0.01	0.01	108.91	0.70	0.49	−0.01	0.02
**Dependent variable: Log of average crossing speed (m/s)**							

*CL trust = Condition level trust, mean of trust for each treatment condition*.

*All six trusting behaviors are measured from the simulation*.

*Fixed effects estimates (β) of the models indicate the direction and degree of relationship between trust and trusting behaviors*.

* *Behaviors with significant relationship with trust*.

**Figure 5:**
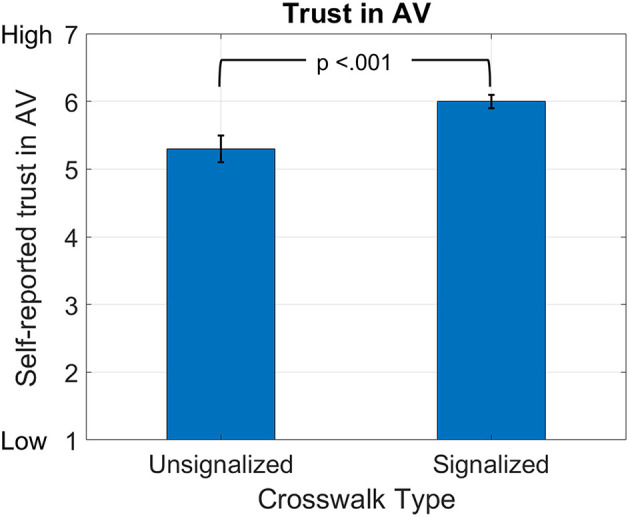
Main effects of signalized crosswalks. Higher self-reported AV trust in signalized conditions.

**Figure 6:**
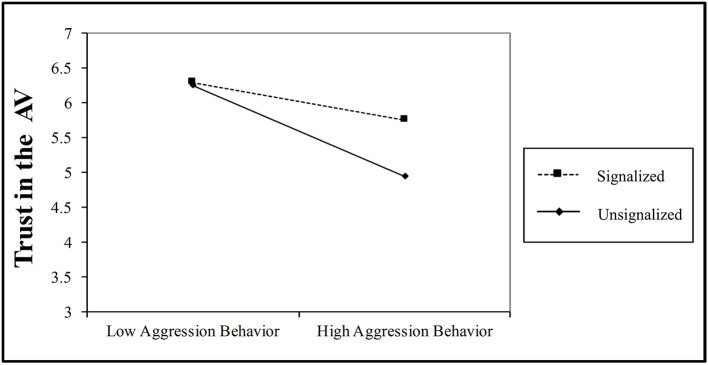
Moderation of aggressive driving by signalized crosswalks. Trust reduction due to high aggression behavior is lower for signalized than unsignalized crosswalks.

H1 posited that aggressive driving decreases trust in the AV; this was supported (fixed effects estimate, β = −0.17, *p* < 0.001). H2, which stated that signalized crosswalk increases trust in the AV, was supported (fixed effects estimate, β = 0.53, *p* < 0.001). Figure 5 shows the main effect of the signalized crosswalk vs. unsignalized crosswalk on trust in the AV. Finally, H3 was examined in the full model (Table 5). H3, the impact of aggressive driving on trust depends on the type of crosswalk, was also supported (fixed effects estimate, β = 0.38, *p* < 0.001) shown in Figure 6.

H4, which stated that trust in the AV leads to more trusting behaviors, was partially supported. We defined trusting behavior as behavior that prolongs a participant's exposure to being vulnerable to the AV's actions. Therefore, when participants trusted the AV, we expected participants to cross closer to AVs resulting in decreased average distance to collision, cross earlier resulting in decreased wait time and overall task time, and walk slowly resulting in decreased crossing speed. We also expected the participants to take more risks and cross when it was not their right-of-way resulting in increased jaywalking time and increased crossing time due to decreased crossing speed. We employed an MLRM for each of these objective measures of trusting behavior with the objective measure being the dependent variable and self-reported trust being the independent variable.

Trust in the AV was significantly related to average distance to collision (fixed effects estimate, β = −0.38, *p* < 0.001), average jaywalking time (fixed effects estimate, β = 0.17, *p* < 0.05), average waiting time (fixed effects estimate, β = 0.18, *p* < 0.001), and overall task time (fixed effects estimate, β = 32.31, *p* < 0.001). In other words, the more participants trusted the AV, the closer they came to the AV while crossing, the more they jaywalked, the longer they waited to cross and more time it took for them to complete the task. Trust in the AV was not related to average crossing time (fixed effects estimate, β = −0.08, *p* > 0.05) or average crossing speed (fixed effects estimate, β = 0.02, *p* > 0.05; see Table 6).

Following previous literature, a lack of visual monitoring of the automation can also be viewed as an act of trusting behavior (Hergeth et al., 2016). Therefore, we expected that trust in AVs would negatively correlate with gaze at AVs. This could be an indication that participants were not concerned about being hit by the AV. To better understand the relationship between monitoring and trust, we divided our analysis by one of three actions: waiting, crossing, and tasking. *Waiting* included the time a participant spent waiting to cross the street. *Crossing* included the actual walking across the street. *Tasking* included the remaining time spent working on the task of moving the balls. We calculate gaze ratios per action by dividing the duration a participant focused on a particular area by the action type's total duration (Table 7). Then, we conducted a repeated measure correlation between each gaze area ratio and trust in the AV (Table 8).

**Table 7:** Gaze distribution by areas of interest (AOI) and driving behavior condition.

**AOI**	**Defensive (%)**	**Normal (%)**	**Aggressive (%)**	**Overall (%)**
AVs approaching the crosswalk	25.0	18.1	13.4	18.7
Checking for AVs (looking in the general direction of AVs when no AVs are present on the road)	2.0	2.3	4.4	2.9
Crosswalk and buildings across the crosswalk	35.9	38.0	40.6	38.4
Task elements (racks on either side of crosswalk)	11.5	12.2	13.2	12.3
Pedestrian signal light on either side of crosswalk	3.4	3.5	3.7	3.6
Traffic light	0.7	0.7	0.4	0.6
All other areas	21.5	25.2	24.3	23.5

**Table 8:** Repeated measures correlation between gaze and trust separated by activity and driving condition.

	**Waiting**			**Crossing**			**Tasking**		
**Areas of interest**	**Defensive**	**Normal**	**Aggressive**	**Defensive**	**Normal**	**Aggressive**	**Defensive**	**Normal**	**Aggressive**
AVs approaching the crosswalk	−0.07	−0.24	−0.13	−0.23	−0.34^*^	−0.25^*^	−0.03	−0.03	0.00
Checking for AVs (looking in the general direction of AVs when no AVs are present on the road)	−0.22	−0.11	−0.13	−0.16	−0.34^*^	−0.24	−0.16	0.13	−0.13
Crosswalk and buildings across the crosswalk	0.24^*^	0.42^***^	0.27^**^	0.13	0.38^**^	0.20	0.19	0.44^***^	0.19
Task elements (racks on either side of crosswalk)	0.01	−0.04	−0.12	0.07	0.05	−0.12	0.00	−0.08	−0.14
Pedestrian signal light on either side of crosswalk^†^	−0.02	−0.03	0.02	−0.13	−0.22^*^	0.05	−0.07	−0.04	0.02
Traffic light^†^	−0.20^*^	−0.14	−0.02	−0.02	0.04	−0.07	−0.20^*^	−0.17	−0.06
All other areas	−0.15	−0.32^*^	−0.31^*^	−0.10	−0.37^**^	−0.19	−0.07	−0.30^*^	−0.14

† *Pedestrian signal light and traffic light AOI available only during the three signalized conditions*.

*A mixed linear model fitted between trust and each gaze ratio to calculate the repeated measures correlation*.

* *Correlation is significant at the 0.05 level (two-tailed)*.

** *Correlation is significant at the 0.01 level (two-tailed)*.

*** *Correlation is significant at the 0.001 level (two-tailed)*.

Trust in the AV was negatively related to monitoring. Time spent looking at the approaching AVs while crossing was negatively correlated with trust in the AV for normal and aggressive driving. In addition, there was negative correlation between self-reported AV trust and time spent checking for AVs while crossing in normal driving behavior. Looking at the pedestrian light while crossing in normal behavior and looking at traffic light while waiting and tasking in defensive behavior were negatively correlated with self-reported AV trust. These results support previous research suggesting that decreased monitoring is related to increased trust (Hergeth et al., 2016). While waiting at the crosswalk, gaze at the crosswalk and the buildings across the crosswalk indicate that the pedestrians were staring ahead and not monitoring the AVs. This time spent looking at the crosswalk and buildings positively correlated with trust.

## Discussion

In this study, we proposed hypotheses for the development of trust based on information availability. When two agents are interacting, the more information gained about the other agent, the less uncertain one is about the other agent. We highlight the importance of AV driving behavior and traffic signal and the moderation effect of traffic signal on the impact of aggressive driving on pedestrians' trust in AVs. Specifically, we found that both sources of information, AV driving behavior and traffic signal, predicted pedestrians' trust in the AVs.

We systematically examined AV driving behavior and found that aggressive AV driving behavior significantly decreased AV trust. Thus, driving behavior could implicitly convey the AV intent to pedestrians. This finding aligns with existing research that has found pedestrians generally prefer a conservative AV driving behavior (Pillai, 2017; Ackermann et al., 2018; Fuest et al., 2018).

Our study also calls attention to the importance of the presence of traffic signals in pedestrian–AV interactions. To the authors' knowledge, impact of traffic signal on pedestrian trust in AVs has not been explored before. We found that pedestrians, in general, trusted the signalized crosswalks more than the unsignalized crosswalks. This is in line with existing research in pedestrian–HDV interactions, which have reported increased trusting behavior such as lower crossing speeds, reduced gaze at vehicles, and shorter distances to collision at signalized crosswalks (Tom and Granie, 2011; Asaithambi et al., 2016; Rasouli et al., 2017).

More importantly, we found that influence of the AV's driving behavior is largely determined by whether the crosswalk is signalized or unsignalized. Signalized crosswalks significantly reduced the negative effects of aggressive driving on trust. It could be because signalized crosswalks dictate the right of way, and AVs are expected to follow the right-of-way (Meeder et al., 2017). Thus, the AVs, irrespective of their driving behavior, are always expected to stop when the pedestrian has the right-of-way. In any case, our findings demonstrate the importance of incorporating the presence of traffic signal when understanding trust in the AV and help to identify generalized situations during which pedestrians trust AVs. For example, trust is generally high in signalized conditions irrespective of the driving behavior (refer to Figure 6).

This study highlights the link between trust in the AV and trusting behaviors. We hypothesized that trust in the AV increases trusting behaviors related to the AV (H4). Our findings related to trusting behaviors fall into three categories. The first category confirms our initial hypothesis. When pedestrians undertrusted the AVs, they exhibited behaviors such as high distance to collision, fewer instances of jaywalking, more looking at AVs while crossing, etc. As trust in the AV increased, pedestrians were much more willing to be vulnerable to the actions of the AV, which came in the form of reductions in distance to collision and increases in jaywalking. We also observed trusting behavior in the form of a lack of monitoring the AVs (i.e., low gaze ratio at the AVs when the self-reported trust scores were high), which aligns with existing research on drivers' trust in AVs (Hergeth et al., 2017). Pedestrians were more willing to place themselves in harm's way when they trusted the AV. However, this behavior can also be unsafe and lead to injury, exemplifying the issue of overtrust. Thus, by examining pedestrian trusting behaviors, our study calls attention to trust calibration of pedestrians for safe interactions with AVs.

However, the second category of trusting behaviors was contrary to our expectations. Increases in trust in the AV led to increases in overall task time and average wait time. One interpretation of our results is that the more pedestrians trusted the AV, the less worried and hurried they were to complete the task. In this sense, increases in overall task time and waiting times might be expected. The third category refutes our hypothesis. Trust in the AV did not have an impact on total crossing time or average crossing time. Similarly, crossing speed was found to be unrelated to trust in the AV. Nevertheless, overall, our study highlights the link between trust and trusting behaviors regarding AVs. Behavioral measures such as distance to collision, jaywalking time, and looking at AVs are more indicative of pedestrian's trust in AVs. This agrees with existing studies that have used similar measures to indicate pedestrian trust (Tom and Granie, 2011; Asaithambi et al., 2016; Rasouli et al., 2017).

The link between certain trusting behavior and self-reported trust identified in this study facilitates real-time measurement of trust in AVs. Measures such as gaze ratio at AVs while waiting and waiting times are trusting behaviors exhibited before the actual start of crossing. These behaviors could be used by the AVs to estimate pedestrians trust in the AVs, which in turn could be used to moderate the driving behavior of AVs to calibrate pedestrian trust in the AVs. For example, if pedestrian trust is estimated to be high, the AV can exhibit an aggressive driving behavior to reduce trust, and when the trust is estimated to be low, the AV can exhibit a defensive driving behavior to improve the trust in the AVs.

Existing studies (Pillai, 2017; Zimmermann and Wettach, 2017) mostly employed predetermined velocity profiles and thus were not reactive to pedestrians. In these studies, vehicles were perceived to be reactive to participants because participants were always placed close to the road at a ready-to-cross position. Our study, however, employed a reactive driving behavior model for the AV based on discrete pedestrian positional states (refer to Table 1). This reactive behavior is more similar to how real-life interactions between vehicles and pedestrians would take place. For example, the AV slows down only when the pedestrian is close to the crosswalk (refer to Table 1) and not when he or she is walking along the sidewalk to reach the crosswalk (refer to paths 3 and 6 in Figure 3A).

Current IVE-based studies are also limited by the range of the possible pedestrian motions (Deb et al., 2017; Pillai, 2017). This limits the number of potential scenarios that can be explored. Our experimental setup with an omnidirectional treadmill provided unlimited range to the pedestrians to walk in the IVE. This allowed us to have a crossing task where participants had an approach distance to the crosswalk in addition to the actual crossing, which is more comparable to real-life crossing situations. Furthermore, the extended range allowed us to examine pedestrian–AV interactions in more complex scenarios with wider roads. Our IVE setup also facilitated study of pedestrian gaze behavior. Our methodology to automatically identify AOI in real time was a precursor to developing algorithms that can identify real-time AV trust through gaze.

## Limitations

This study has several limitations. First, the AV behavior models were based only on discrete states driven by the pedestrian's position and did not incorporate continuous dynamics. Moreover, pedestrian intent was derived from only their position and did not consider their orientation. Second, we conducted our study in an IVE, which is a controlled experimental setting. Owing to the presence of other environmental and situational factors, participants might react differently in an actual crosswalk, resulting in different trusting behaviors. However, some evidence suggests that this is not the case (Heydarian et al., 2015; Deb et al., 2017). Specifically, Deb et al. (2017) found that pedestrians' reactions to traffic situations in an IVE were similar to those in the real world. Nonetheless, we acknowledge this as a potential limitation.

Third, our study only examined fully automated vehicles without humans. It is unclear whether our findings can be generalized to AVs with safety drivers or partially automated vehicles. Fourth, our study considered only one kind of an AV, a sedan. We acknowledge that there may be behavioral differences due to the size and type of the AV (de Clercq et al., 2019). Fifth, although we employed a gaze analysis methodology that enables automatic AOI identification in real time, it does not segregate fixations from saccades. Fixations indicate the steady gaze focused on a particular region, whereas saccades represent rapid movements between the fixations. Future work should consider incorporating fixations into the methodology. Sixth, our study design involved only one human participant on a unidirectional street. Future studies could include more pedestrians, different road layouts, and bidirectional streets. Finally, our participants were all young university students who might have a similar attitude toward AVs. Future studies might enlist representatives from the general population or older individuals whose trust or acceptance of AVs could vary significantly (Schoettle and Sivak, 2014; Hulse et al., 2018).

## Conclusion and Future Work

We formally examined the effects of implicit AV communication through driving behavior and traffic signal on pedestrian trust in AVs. We examined the moderation effects of traffic signal on the impact of AV driving behavior on pedestrian trust in AVs. We also established the relationships between trust and trusting behaviors. Pedestrians trusted the AVs more when the AVs exhibited defensive driving behavior. Furthermore, pedestrians trusted AVs more at signalized crosswalks, and this trust was unaffected by the driving behavior of the AVs. This study thus revealed significant relationships among AV driving behavior, crosswalk type, pedestrian's trust in the AVs, and trusting behavior.

Nonetheless, there is still much to learn. For example, under low trust situations (such as aggressive AV driving at unsignalized crosswalks), ways to promote trust through other communication means could be explored. Using an explicit communication interface is one way to provide AV intent information that can reduce the uncertainty and promote AV trust. Furthermore, the relative influence of the two sources of information—AV explicit interface and traffic signal—on pedestrian trust can be examined.

In addition, the correlation between trust and observable trusting behavior opens many avenues for research. Trust is not readily observable. However, by establishing a correlation between trust and observable trusting behavior, trusting behavior can be used as a proxy for measuring trust. This would enable real-time assessment of trust under different conditions. Further research on real-time trust measurement would enable us to develop prediction models of pedestrian trust in AVs based on various pedestrian, vehicle, situational, and environmental factors. Future research can also aim to validate the findings from this study in a real-world setting using Wizard of Oz AVs.

## Data Availability Statement

All datasets generated for this study are included in the article/Supplementary Material.

## Ethics Statement

This study was carried out in accordance with the recommendations of American Psychological Association Code of Ethics and the Institutional Review Board at the University of Michigan with written informed consent from all subjects. All subjects gave written informed consent in accordance with the Declaration of Helsinki. The protocol was approved by the Institutional Review Board at the University of Michigan.

## Author Contributions

SJ, CC, DT, XY, AP, KT, and LR contributed to the conception and design of the study. SJ and CC contributed to the experiment execution. SJ and LR contributed to the analysis. SJ, DT, XY, AP, KT, and LR wrote sections of the manuscript and contributed to manuscript revision.

### Conflict of Interest

KT is an employee of TRI. The remaining authors declare that the research was conducted in the absence of any commercial or financial relationships that could be construed as a potential conflict of interest.

## Footnotes

^1^https://www.nbcnews.com/tech/innovation/humans-harass-attack-self-driving-waymo-cars-n950971

^2^https://drive.google.com/drive/u/1/folders/1jvKOELsDp8Bj4ScJcOmPpKMGZeRosycy

## Appendix

### 1. Latin Square Design

The standard Latin square design that we employed in the study is given below, with the six treatment conditions: defensive unsignalized (A), normal unsignalized (B), aggressive unsignalized (C), defensive signalized (D), normal signalized (E), and aggressive signalized (F). Each condition appears exactly once in each row and once in each column, which resulted in a set of six condition sequences. The set was designed such that every treatment condition appears exactly once before and once after every other condition. The set was repeated five times to get 30 condition sequences for the 30 participants in the study.

**Table A1:** Latin square design employed in the study.

	**Order of treatment conditions**					
**Subject number**	**1st**	**2nd**	**3rd**	**4th**	**5th**	**6th**
1	A	F	B	E	C	D
2	B	A	C	F	D	E
3	C	B	D	A	E	F
4	D	C	E	B	F	A
5	E	D	F	C	A	B
6	F	E	A	D	B	C

### 2. Post-Treatment Trust Questionnaire

The below questionnaire has been adapted from Muir (1987), which examines trust in automation. Please indicate the extent to which you believe the autonomy has each of the following traits (from 1 representing “none at all” to 7 representing “extremely high”).

- Competence: To what extent did the autonomous cars perform their function properly i.e., recognizing you and reacting for you?
- Predictability: To what extent were you able to predict the behavior of the autonomous cars from moment to moment?
- Dependability: To what extent can you count on the autonomous cars to do its job?
- Responsibility: To what extent the autonomous cars seemed to be wary of their surroundings?
- Reliability over time: To what extent do you think the autonomous car's actions were consistent through out the interaction?
- Faith: What degree of faith do you have that the autonomous cars will be able to cope with all uncertain ties in the future?

### 3. Simulator Sickness Questionnaire (SSQ)

Please indicate how much each symptom below is affecting you right now [survey from Kennedy et al. (1993)].

**Table A2:** Simulator sickness questionnaire.

**Sensation**	**0**	**1**	**2**	**3**
General Discomfort	None	Slight	Moderate	Severe
Fatigue	None	Slight	Moderate	Severe
Headache	None	Slight	Moderate	Severe
Eye Strain	None	Slight	Moderate	Severe
Difficulty focusing	None	Slight	Moderate	Severe
Increased salivation	None	Slight	Moderate	Severe
Sweating	None	Slight	Moderate	Severe
Nausea	None	Slight	Moderate	Severe
Difficulty concentrating	None	Slight	Moderate	Severe
Fullness of head	None	Slight	Moderate	Severe
Blurred vision	None	Slight	Moderate	Severe
Dizzy (eyes open)	None	Slight	Moderate	Severe
Dizzy (eyes closed)	None	Slight	Moderate	Severe
Vertigo	None	Slight	Moderate	Severe
Stomach awareness	None	Slight	Moderate	Severe
Burping	None	Slight	Moderate	Severe
