# Supplementary material for: Pedestrian Trust in Automated Vehicles: Role of Traffic Signal and AV Driving Behavior
Source: Front Robot AI. 2019 Nov 28;6:117. doi: 10.3389/frobt.2019.00117 (PMC7805667; doi:10.3389/frobt.2019.00117)
Supplement: Supplementary file 1 [file Table_1.docx]

>*********************************Variables Code Key*******************************

SubjectID - Subject identification number (1-30)

ScenarioID - Treatment condition identification number (1-6); 1- Defensive Unsignalized, 2 - Normal Unsignalized, 3 - Aggressive Unsignalized, 4- Defensive Signalized, 5 - Normal Signalized, 6 - Aggressive Signalized

ScenarioName - Corresponding Treatment condition name

Gender

DrivingCondition - One of three driving conditions (1 - Defensive, 2 - Normal, 3 - Aggressive)

SignalCondition - One of two signal conditions (1 - Unsignalzied, 2 - Signalzied)

Age - (in years)

TrustPropensity - Propensity to Trust; Mean of responses to the 'Complacency rating Scale'

VRexperiencescore - Prior experience with Virtual Reality; Mean of responses to VR questions in 'Pre-experiment Survey'

Disorientationscore - Calculated from 'Simulation Sickness Questionnaire'; A factor of SSQ

Nauseascore - Calculated from 'Simulation Sickness Questionnaire'; A factor of SSQ

Oculomotorscore - Calculated from 'Simulation Sickness Questionnaire'; A factor of SSQ

SSQScore - Simulation Sickness Score; Calculated from Disorientation, Nausea and Oculomotor scores

OverallTrustScore - Self-reported trust in Automated Vehicles (AVs); Mean of responses to Trust Questionnaire

CLTrust - Condition Level Trust (Mean of Overall Trust Score of each treatment condition)

Perceivedaggression - Self-reported perceived driving aggression of the AV

Averagecrossingspeedms - Mean pedestrian speed for the duration of crossing over all six crossings for a treatment condition

Averagecrossingtimes - Mean duration of crossing over all six crossings for a treatment condition

Averagewaitingtimes - Mean duration spent waiting at the crosswalk before crossing over all six crossings for a treatment condition

Averagejaywalktimes - Mean duration spent jaywalking over all six crossings for a treatment condition

Averagedistancetocollisionm - Mean distance between pedestrian and vehicle when the pedestrian enters the crosswalk over all six crossings for a treatment condition

Overalltasktimes - Mean duration spent in the treatment condition

WaitRatio_traffic - Ratio of duration spent looking at traffic signal over the entire duration when the subject is waiting to cross

WaitRatio_task - Ratio of duration spent looking at task elements (racks) over the entire duration when the subject is waiting to cross

WaitRatio_untagged - Ratio of duration spent looking at other items in the environment over the entire duration when the subject is waiting to cross

WaitRatio_check_vehicle - Ratio of duration spent looking in the general direction of the vehicle over the entire duration when the subject is waiting to cross

WaitRatio_Signal - Ratio of duration spent looking at the pedestrian signals at either side over the entire duration when the subject is waiting to cross

WaitRatio_look_vehicle - Ratio of duration spent looking at the AVs over the entire duration when the subject is waiting to cross

WaitRatio_crosswalk - Ratio of duration spent looking at the crosswalk and the buildings across the crosswalk over the entire duration when the subject is waiting to cross

CrossRatio_traffic - Ratio of duration spent looking at traffic signal over the entire duration when the subject is crossing

CrossRatio_task - Ratio of duration spent looking at task elements (racks) over the entire duration when the subject is crossing

CrossRatio_untagged - Ratio of duration spent looking at other items in the environment over the entire duration when the subject is crossing

CrossRatio_check_vehicle - Ratio of duration spent looking in the general direction of the vehicle over the entire duration when the subject is crossing

CrossRatio_Signal - Ratio of duration spent looking at the pedestrian signals at either side over the entire duration when the subject is crossing

CrossRatio_look_vehicle - Ratio of duration spent looking at the AVs over the entire duration when the subject is crossing

CrossRatio_crosswalk - Ratio of duration spent looking at the crosswalk and the buildings across the crosswalk over the entire duration when the subject is crossing

TaskRatio_traffic - Ratio of duration spent looking at traffic signal over the entire duration when the subject is walking on the sidewalk and performing the task

TaskRatio_task - Ratio of duration spent looking at task elements (racks) over the entire duration when the subject is walking on the sidewalk and performing the task

TaskRatio_untagged - Ratio of duration spent looking at other items in the environment over the entire duration when the subject is walking on the sidewalk and performing the task

TaskRatio_check_vehicle - Ratio of duration spent looking in the general direction of the vehicle over the entire duration when the subject is walking on the sidewalk and performing the task

TaskRatio_Signal - Ratio of duration spent looking at the pedestrian signals at either side over the entire duration when the subject is walking on the sidewalk and performing the task

TaskRatio_look_vehicle - Ratio of duration spent looking at the AVs over the entire duration when the subject is walking on the sidewalk and performing the task

TaskRatio_crosswalk - Ratio of duration spent looking at the crosswalk and the buildings across the crosswalk over the entire duration when the subject is walking on the sidewalk and performing the task

Log10DTC - Log of Mean distance between pedestrian and vehicle when the pedestrian enters the crosswalk over all six crossings for a treatment condition

Log10WaitTime - Log of Mean duration spent waiting at the crosswalk before crossing over all six crossings for a treatment condition

Log10JaywalkTime - Log of Mean duration spent jaywalking over all six crossings for a treatment condition

ZTrustForDrivingBehavior - Normalized Overall trust within each driving behavior

>*******************************************************************************************************************************

DATASET ACTIVATE DataSet1.

>***********5.1 Manipulation Check (Refer Page 9 in Manuscript)***************************************

MIXED Perceivedaggression BY DrivingCondition

/CRITERIA=CIN(95) MXITER(100) MXSTEP(10) SCORING(1) SINGULAR(0.000000000001) HCONVERGE(0,

ABSOLUTE) LCONVERGE(0, ABSOLUTE) PCONVERGE(0.000001, ABSOLUTE)

/FIXED=DrivingCondition | SSTYPE(3)

/METHOD=REML

/PRINT=CORB SOLUTION

/REPEATED=ScenarioID | SUBJECT(SubjectID) COVTYPE(DIAG)

/EMMEANS=TABLES(DrivingCondition) COMPARE ADJ(LSD).

>**************************************************************************************************************

>**************************************************************************************************************

>***********************5.2 Measurement Validity (Refer Page 9 in Manuscript )***************************************

>***********Factor Analysis (Refer Page 9 and Table 3 in Page 20 in Manuscript )***************************************

FACTOR

/VARIABLES Competence Predictability Dependability Responsibility Reliability Faith Disorientationscore Nauseascore Oculomotorscore

/MISSING LISTWISE

/ANALYSIS Competence Predictability Dependability Responsibility Reliability Faith Disorientationscore Nauseascore Oculomotorscore

/PRINT INITIAL CORRELATION KMO AIC EXTRACTION ROTATION FSCORE

/CRITERIA MINEIGEN(1) ITERATE(25)

/EXTRACTION PC

/CRITERIA ITERATE(25)

/ROTATION VARIMAX

/METHOD=CORRELATION.

>**************************************************************************************************************

>****************************************** Gjejser test for heteroscedasticity**************************************************************************

>*********************** Step 1 - Regression of trust with independent objective variables*****************************

REGRESSION

/MISSING LISTWISE

/STATISTICS COEFF OUTS R ANOVA

/CRITERIA=PIN(.05) POUT(.10)

/NOORIGIN

/DEPENDENT CLTrust

/METHOD=ENTER Averagecrossingspeedms Averagecrossingtimes Averagewaitingtimes Averagejaywalktimes

Averagedistancetocollisionm Overalltasktimes

/SAVE RESID.

>*********************** Step 2 - Calculate Absolute value of residuals*****************************

COMPUTE AbsUt=ABS(RES_1).

EXECUTE.

>*********************** Step 3 - Regression of Absolute value of residuals*****************************

REGRESSION

/MISSING LISTWISE

/STATISTICS COEFF OUTS R ANOVA

/CRITERIA=PIN(.05) POUT(.10)

/NOORIGIN

/DEPENDENT AbsUt

/METHOD=ENTER Averagecrossingspeedms Averagecrossingtimes Averagewaitingtimes Averagejaywalktimes

Averagedistancetocollisionm Overalltasktimes.

>*****************Step 4 - Log transformation to reduce unequal variances and ensure homoscedasticity*****************************

COMPUTE Log10DTC=LG10(Averagedistancetocollisionm).

EXECUTE.

COMPUTE Log10WaitTime=LG10(Averagewaitingtimes).

EXECUTE.

COMPUTE Log10JaywalkTime=LG10(Averagejaywalktimes).

EXECUTE.

>*********************** Step 5 - Regression of trust with transformed independent objective variables*****************************

REGRESSION

/MISSING LISTWISE

/STATISTICS COEFF OUTS R ANOVA

/CRITERIA=PIN(.05) POUT(.10)

/NOORIGIN

/DEPENDENT CLTrust

/METHOD=ENTER Log10JaywalkTime Log10CrossingSpeed Overalltasktimes Averagecrossingtimes Averagewaitingtimes Log10DTC

/SAVE RESID.

>*********************** Step 6 - Calculate Absolute value of residuals*****************************

COMPUTE LogAbsUt=ABS(RES_2).

EXECUTE.

>*********************** Step 7 - Regression of Absolute value of residuals with transformed variables*****************************

REGRESSION

/MISSING LISTWISE

/STATISTICS COEFF OUTS R ANOVA

/CRITERIA=PIN(.05) POUT(.10)

/NOORIGIN

/DEPENDENT LogAbsUt

/METHOD=ENTER Log10JaywalkTime Log10CrossingSpeed Overalltasktimes Averagecrossingtimes Averagewaitingtimes Log10DTC.

>****************************************************************************************************************************************

>****************************************************************************************************************************************

>*******************************5.3 Population Effects on Trust (Refer Page 10 in Manuscript)*********************

>***********************Gender effects on Trust************************************************

MIXED OverallTrustScore BY Gender

/CRITERIA=CIN(95) MXITER(100) MXSTEP(10) SCORING(1) SINGULAR(0.000000000001) HCONVERGE(0,

ABSOLUTE) LCONVERGE(0, ABSOLUTE) PCONVERGE(0.000001, ABSOLUTE)

/FIXED=Gender | SSTYPE(3)

/METHOD=REML

/PRINT=CORB COVB G SOLUTION TESTCOV

/RANDOM=INTERCEPT | SUBJECT(SubjectID) COVTYPE(VC)

/EMMEANS=TABLES(OVERALL).

>**********************************************************************

>***********************Age effects on Trust************************************************

MIXED OverallTrustScore WITH ZAge

/CRITERIA=CIN(95) MXITER(100) MXSTEP(10) SCORING(1) SINGULAR(0.000000000001) HCONVERGE(0,

ABSOLUTE) LCONVERGE(0, ABSOLUTE) PCONVERGE(0.000001, ABSOLUTE)

/FIXED=ZAge | SSTYPE(3)

/METHOD=REML

/PRINT=CORB COVB G SOLUTION TESTCOV

/RANDOM=INTERCEPT | SUBJECT(SubjectID) COVTYPE(VC)

/EMMEANS=TABLES(OVERALL).

>**********************************************************************************************************************************************

>**********************************************************************************************************************************************

>*******************************5.4 Hypotheses Testing (Refer Page 10 in Manuscript)*********************

>********************************Hypothesis 1: Aggressive Driving reduces trust**********************************

MIXED OverallTrustScore WITH ZPerceivedaggression

/CRITERIA=CIN(95) MXITER(100) MXSTEP(10) SCORING(1) SINGULAR(0.000000000001) HCONVERGE(0,

ABSOLUTE) LCONVERGE(0, ABSOLUTE) PCONVERGE(0.000001, ABSOLUTE)

/FIXED=ZPerceivedaggression | SSTYPE(3)

/METHOD=REML

/PRINT=CORB SOLUTION TESTCOV

/REPEATED=ScenarioID | SUBJECT(SubjectID) COVTYPE(DIAG).

>**********************************************************************************************************************************************

>********************************Hypothesis 2: Signalized crosswalks increases trust**********************************

MIXED OverallTrustScore WITH SignalCondition

/CRITERIA=CIN(95) MXITER(100) MXSTEP(10) SCORING(1) SINGULAR(0.000000000001) HCONVERGE(0,

ABSOLUTE) LCONVERGE(0, ABSOLUTE) PCONVERGE(0.000001, ABSOLUTE)

/FIXED=SignalCondition | SSTYPE(3)

/METHOD=REML

/PRINT=CORB SOLUTION TESTCOV

/REPEATED=ScenarioID | SUBJECT(SubjectID) COVTYPE(DIAG).

>**********************************************************************************************************************************************

>********************************Hypothesis 3: Effect of aggressive driving moderated by Signal Condition (Refer Table 4 in Page 21)**********************************

MIXED OverallTrustScore BY SignalCondition WITH ZPerceivedaggression

/CRITERIA=CIN(95) MXITER(100) MXSTEP(10) SCORING(1) SINGULAR(0.000000000001) HCONVERGE(0,

ABSOLUTE) LCONVERGE(0, ABSOLUTE) PCONVERGE(0.000001, ABSOLUTE)

/FIXED=ZPerceivedaggression SignalCondition SignalCondition*ZPerceivedaggression | SSTYPE(3)

/METHOD=REML

/PRINT=CORB COVB SOLUTION TESTCOV

/REPEATED=ScenarioID | SUBJECT(SubjectID) COVTYPE(DIAG).

>**********************************************************************************************************************************************

>******************************Model Comparison BIC values (Refer Page 10 in Manuscript)*********************************************

>***************************No interaction term, Maximum Likelihood estimation**************************

MIXED OverallTrustScore WITH ZPerceivedaggression ZAge

ZTrustPropensity ZVRexperiencescore ZSSQScore DrivingCondition SignalCondition

/CRITERIA=CIN(95) MXITER(100) MXSTEP(10) SCORING(1) SINGULAR(0.000000000001) HCONVERGE(0,

ABSOLUTE) LCONVERGE(0, ABSOLUTE) PCONVERGE(0.000001, ABSOLUTE)

/FIXED=DrivingCondition SignalCondition ZPerceivedaggression ZAge ZTrustPropensity

ZVRexperiencescore ZSSQScore| SSTYPE(3)

/METHOD=ML

/PRINT=CPS CORB COVB DESCRIPTIVES G LMATRIX R SOLUTION TESTCOV

/RANDOM=INTERCEPT DrivingCondition SignalCondition | SUBJECT(SubjectID) COVTYPE(VC)

/REPEATED=ScenarioID | SUBJECT(SubjectID) COVTYPE(DIAG).

>******************************With interaction term, and random effects*********************************************

MIXED OverallTrustScore WITH ZPerceivedaggression ZAge ZTrustPropensity ZVRexperiencescore

ZSSQScore DrivingCondition SignalCondition

/CRITERIA=CIN(95) MXITER(100) MXSTEP(10) SCORING(1) SINGULAR(0.000000000001) HCONVERGE(0,

ABSOLUTE) LCONVERGE(0, ABSOLUTE) PCONVERGE(0.000001, ABSOLUTE)

/FIXED=ZPerceivedaggression SignalCondition ZPerceivedaggression*SignalCondition DrivingCondition ZAge ZTrustPropensity ZVRexperiencescore ZSSQScore

| SSTYPE(3)

/METHOD=REML

/PRINT=CORB COVB G LMATRIX R SOLUTION TESTCOV

/RANDOM=INTERCEPT DrivingCondition SignalCondition | SUBJECT(SubjectID) COVTYPE(VC)

/REPEATED=ScenarioID | SUBJECT(SubjectID) COVTYPE(DIAG).

>**********************************************************************************************************************************************

>******Table 4. Trust Model: Aggressive Driving and Signal Condition Influenced Trust 5 (Refer Page 21 in Manuscript)***************

>*****************************************************************************************

MIXED OverallTrustScore WITH ZPerceivedaggression DrivingCondition SignalCondition ZAge ZTrustPropensity ZVRexperiencescore ZSSQScore

/CRITERIA=CIN(95) MXITER(100) MXSTEP(10) SCORING(1) SINGULAR(0.000000000001) HCONVERGE(0,

ABSOLUTE) LCONVERGE(0, ABSOLUTE) PCONVERGE(0.000001, ABSOLUTE)

/FIXED=ZPerceivedaggression SignalCondition ZPerceivedaggression*SignalCondition DrivingCondition ZAge ZTrustPropensity ZVRexperiencescore ZSSQScore | SSTYPE(3)

/METHOD=REML

/PRINT=CORB SOLUTION TESTCOV

/REPEATED=ScenarioID | SUBJECT(SubjectID) COVTYPE(DIAG).

>**********************************************************************************************************************************************

>**********************Table 5.Correlations of Main Variables (Refer Page 21 in Manuscript)*************************************

CORRELATIONS

/VARIABLES=OverallTrustScore Perceivedaggression SignalCondition DrivingCondition Age TrustPropensity VRexperiencescore SSQScore

/PRINT=TWOTAIL NOSIG

/MISSING=PAIRWISE.

>**************************************************************************************************

>*************************Table 6. Trust Predicting Trusting Behaviors (Refer Page 22 in Manuscript)********************************

>**************************Mixed Linear Model of Average Distance to Collision regressed on Condition Level Trust*************************

MIXED Log10DTC WITH ZCLTrust

/CRITERIA=CIN(95) MXITER(100) MXSTEP(10) SCORING(1) SINGULAR(0.000000000001) HCONVERGE(0,

ABSOLUTE) LCONVERGE(0, ABSOLUTE) PCONVERGE(0.000001, ABSOLUTE)

/FIXED=ZCLTrust | SSTYPE(3)

/METHOD=REML

/PRINT=CORB SOLUTION TESTCOV

/REPEATED=ScenarioID | SUBJECT(SubjectID) COVTYPE(DIAG).

>****************************************************************************************************

>***********************Mixed Linear Model of Average Jaywalking Time regressed on Condition Level Trust **********************************

MIXED Log10JaywalkTime WITH ZCLTrust

/CRITERIA=CIN(95) MXITER(100) MXSTEP(10) SCORING(1) SINGULAR(0.000000000001) HCONVERGE(0,

ABSOLUTE) LCONVERGE(0, ABSOLUTE) PCONVERGE(0.000001, ABSOLUTE)

/FIXED=ZCLTrust | SSTYPE(3)

/METHOD=REML

/PRINT=CORB SOLUTION TESTCOV

/REPEATED=ScenarioID | SUBJECT(SubjectID) COVTYPE(DIAG).

>***************************************************************************************************

>***********************Mixed Linear Model of Average Watiting Time regressed on Condition Level Trust ***********************************

MIXED Averagewaitingtimes WITH ZCLTrust

/CRITERIA=CIN(95) MXITER(100) MXSTEP(10) SCORING(1) SINGULAR(0.000000000001) HCONVERGE(0,

ABSOLUTE) LCONVERGE(0, ABSOLUTE) PCONVERGE(0.000001, ABSOLUTE)

/FIXED=ZCLTrust | SSTYPE(3)

/METHOD=REML

/PRINT=CORB SOLUTION TESTCOV

/REPEATED=ScenarioID | SUBJECT(SubjectID) COVTYPE(DIAG).

>************************************************************************************************

>**************************Mixed Linear Model of Overall Task Time regressed on Condition Level Trust *********************************************

MIXED Overalltasktimes WITH ZCLTrust

/CRITERIA=CIN(95) MXITER(100) MXSTEP(10) SCORING(1) SINGULAR(0.000000000001) HCONVERGE(0,

ABSOLUTE) LCONVERGE(0, ABSOLUTE) PCONVERGE(0.000001, ABSOLUTE)

/FIXED=ZCLTrust | SSTYPE(3)

/METHOD=REML

/PRINT=CORB SOLUTION TESTCOV

/REPEATED=ScenarioID | SUBJECT(SubjectID) COVTYPE(DIAG).

>************************************************************************************************

>**************************Mixed Linear Model of Average Crossing Time regressed on Condition Level Trust***********************************

MIXED Averagecrossingtimes WITH ZCLTrust

/CRITERIA=CIN(95) MXITER(100) MXSTEP(10) SCORING(1) SINGULAR(0.000000000001) HCONVERGE(0,

ABSOLUTE) LCONVERGE(0, ABSOLUTE) PCONVERGE(0.000001, ABSOLUTE)

/FIXED=ZCLTrust | SSTYPE(3)

/METHOD=REML

/PRINT=CORB SOLUTION TESTCOV

/REPEATED=ScenarioID | SUBJECT(SubjectID) COVTYPE(DIAG).

>************************************************************************************************

>**************************Mixed Linear Model of Average Crossing Speed regressed on Condition Level Trust **********************************

MIXED Log10CrossingSpeed WITH ZCLTrust

/CRITERIA=CIN(95) MXITER(100) MXSTEP(10) SCORING(1) SINGULAR(0.000000000001) HCONVERGE(0,

ABSOLUTE) LCONVERGE(0, ABSOLUTE) PCONVERGE(0.000001, ABSOLUTE)

/FIXED=ZCLTrust | SSTYPE(3)

/METHOD=REML

/PRINT=CORB SOLUTION TESTCOV

/REPEATED=ScenarioID | SUBJECT(SubjectID) COVTYPE(DIAG).

>***********************************************************************************************

>******************************Table 8: Gaze Correlations with Overall trust (Refer Page 23 in Manuscript)*********************************************

SORT CASES BY DrivingCondition.

SPLIT FILE LAYERED BY DrivingCondition.

>********************************************************Gaze correlatins during Waiting********************************************************************

>******************************Overall trust Correlations with gaze ratio of looking at vehicle during waiting*********************************************

MIXED ZTrustForDrivingBehavior WITH ZWaitRatio_look_vehicle

/CRITERIA=CIN(95) MXITER(100) MXSTEP(10) SCORING(1) SINGULAR(0.000000000001) HCONVERGE(0,

ABSOLUTE) LCONVERGE(0, ABSOLUTE) PCONVERGE(0.000001, ABSOLUTE)

/FIXED=ZWaitRatio_look_vehicle | SSTYPE(3)

/METHOD=REML

/PRINT=CORB COVB G SOLUTION TESTCOV

/RANDOM=INTERCEPT | SUBJECT(SubjectID) COVTYPE(VC)

/REPEATED=ScenarioID | SUBJECT(SubjectID) COVTYPE(DIAG)

/EMMEANS=TABLES(OVERALL).

>******************************Overall trust Correlations with gaze ratio of checking for vehicle during waiting*********************************************

MIXED ZTrustForDrivingBehavior WITH ZWaitRatio_check_vehicle

/CRITERIA=CIN(95) MXITER(100) MXSTEP(10) SCORING(1) SINGULAR(0.000000000001) HCONVERGE(0,

ABSOLUTE) LCONVERGE(0, ABSOLUTE) PCONVERGE(0.000001, ABSOLUTE)

/FIXED=ZWaitRatio_check_vehicle | SSTYPE(3)

/METHOD=REML

/PRINT=CORB COVB G SOLUTION TESTCOV

/RANDOM=INTERCEPT | SUBJECT(SubjectID) COVTYPE(VC)

/REPEATED=ScenarioID | SUBJECT(SubjectID) COVTYPE(DIAG)

/EMMEANS=TABLES(OVERALL).

>******************************Overall trust Correlations with gaze ratio of looking at crosswalk during waiting*********************************************

MIXED ZTrustForDrivingBehavior WITH ZWaitRatio_crosswalk

/CRITERIA=CIN(95) MXITER(100) MXSTEP(10) SCORING(1) SINGULAR(0.000000000001) HCONVERGE(0,

ABSOLUTE) LCONVERGE(0, ABSOLUTE) PCONVERGE(0.000001, ABSOLUTE)

/FIXED=ZWaitRatio_crosswalk | SSTYPE(3)

/METHOD=REML

/PRINT=CORB COVB G SOLUTION TESTCOV

/RANDOM=INTERCEPT | SUBJECT(SubjectID) COVTYPE(VC)

/REPEATED=ScenarioID | SUBJECT(SubjectID) COVTYPE(DIAG)

/EMMEANS=TABLES(OVERALL).

>******************************Overall trust Correlations with gaze ratio of looking at traffic signal during waiting*********************************************

MIXED ZTrustForDrivingBehavior WITH ZWaitRatio_traffic

/CRITERIA=CIN(95) MXITER(100) MXSTEP(10) SCORING(1) SINGULAR(0.000000000001) HCONVERGE(0,

ABSOLUTE) LCONVERGE(0, ABSOLUTE) PCONVERGE(0.000001, ABSOLUTE)

/FIXED=ZWaitRatio_traffic | SSTYPE(3)

/METHOD=REML

/PRINT=CORB COVB G SOLUTION TESTCOV

/RANDOM=INTERCEPT | SUBJECT(SubjectID) COVTYPE(VC)

/REPEATED=ScenarioID | SUBJECT(SubjectID) COVTYPE(DIAG)

/EMMEANS=TABLES(OVERALL).

>******************************Overall trust Correlations with gaze ratio of looking at task elements during waiting*********************************************

MIXED ZTrustForDrivingBehavior WITH ZWaitRatio_task

/CRITERIA=CIN(95) MXITER(100) MXSTEP(10) SCORING(1) SINGULAR(0.000000000001) HCONVERGE(0,

ABSOLUTE) LCONVERGE(0, ABSOLUTE) PCONVERGE(0.000001, ABSOLUTE)

/FIXED=ZWaitRatio_task | SSTYPE(3)

/METHOD=REML

/PRINT=CORB COVB G SOLUTION TESTCOV

/RANDOM=INTERCEPT | SUBJECT(SubjectID) COVTYPE(VC)

/REPEATED=ScenarioID | SUBJECT(SubjectID) COVTYPE(DIAG)

/EMMEANS=TABLES(OVERALL).

>******************************Overall trust Correlations with gaze ratio of looking at Pedestrian Signal during waiting*********************************************

MIXED ZTrustForDrivingBehavior WITH ZWaitRatio_Signal

/CRITERIA=CIN(95) MXITER(100) MXSTEP(10) SCORING(1) SINGULAR(0.000000000001) HCONVERGE(0,

ABSOLUTE) LCONVERGE(0, ABSOLUTE) PCONVERGE(0.000001, ABSOLUTE)

/FIXED=ZWaitRatio_Signal | SSTYPE(3)

/METHOD=REML

/PRINT=CORB COVB G SOLUTION TESTCOV

/RANDOM=INTERCEPT | SUBJECT(SubjectID) COVTYPE(VC)

/REPEATED=ScenarioID | SUBJECT(SubjectID) COVTYPE(DIAG)

/EMMEANS=TABLES(OVERALL).

>******************************Overall trust Correlations with gaze ratio of looking at other areas in environment during waiting*********************************************

MIXED ZTrustForDrivingBehavior WITH ZWaitRatio_untagged

/CRITERIA=CIN(95) MXITER(100) MXSTEP(10) SCORING(1) SINGULAR(0.000000000001) HCONVERGE(0,

ABSOLUTE) LCONVERGE(0, ABSOLUTE) PCONVERGE(0.000001, ABSOLUTE)

/FIXED=ZWaitRatio_untagged | SSTYPE(3)

/METHOD=REML

/PRINT=CORB COVB G SOLUTION TESTCOV

/RANDOM=INTERCEPT | SUBJECT(SubjectID) COVTYPE(VC)

/REPEATED=ScenarioID | SUBJECT(SubjectID) COVTYPE(DIAG)

/EMMEANS=TABLES(OVERALL).

>********************************************************Gaze correlatins during Crossing********************************************************************

>******************************Overall trust Correlations with gaze ratio of looking at vehicle during crossing*********************************************

MIXED ZTrustForDrivingBehavior WITH ZCrossRatio_look_vehicle

/CRITERIA=CIN(95) MXITER(100) MXSTEP(10) SCORING(1) SINGULAR(0.000000000001) HCONVERGE(0,

ABSOLUTE) LCONVERGE(0, ABSOLUTE) PCONVERGE(0.000001, ABSOLUTE)

/FIXED=ZCrossRatio_look_vehicle | SSTYPE(3)

/METHOD=REML

/PRINT=CORB COVB G SOLUTION TESTCOV

/RANDOM=INTERCEPT | SUBJECT(SubjectID) COVTYPE(VC)

/REPEATED=ScenarioID | SUBJECT(SubjectID) COVTYPE(DIAG)

/EMMEANS=TABLES(OVERALL).

>******************************Overall trust Correlations with gaze ratio of checking for vehicle during crossing*********************************************

MIXED ZTrustForDrivingBehavior WITH ZCrossRatio_check_vehicle

/CRITERIA=CIN(95) MXITER(100) MXSTEP(10) SCORING(1) SINGULAR(0.000000000001) HCONVERGE(0,

ABSOLUTE) LCONVERGE(0, ABSOLUTE) PCONVERGE(0.000001, ABSOLUTE)

/FIXED=ZCrossRatio_check_vehicle | SSTYPE(3)

/METHOD=REML

/PRINT=CORB COVB G SOLUTION TESTCOV

/RANDOM=INTERCEPT | SUBJECT(SubjectID) COVTYPE(VC)

/REPEATED=ScenarioID | SUBJECT(SubjectID) COVTYPE(DIAG)

/EMMEANS=TABLES(OVERALL).

>******************************Overall trust Correlations with gaze ratio of looking at crosswalk during crossing*********************************************

MIXED ZTrustForDrivingBehavior WITH ZCrossRatio_crosswalk

/CRITERIA=CIN(95) MXITER(100) MXSTEP(10) SCORING(1) SINGULAR(0.000000000001) HCONVERGE(0,

ABSOLUTE) LCONVERGE(0, ABSOLUTE) PCONVERGE(0.000001, ABSOLUTE)

/FIXED=ZCrossRatio_crosswalk | SSTYPE(3)

/METHOD=REML

/PRINT=CORB COVB G SOLUTION TESTCOV

/RANDOM=INTERCEPT | SUBJECT(SubjectID) COVTYPE(VC)

/REPEATED=ScenarioID | SUBJECT(SubjectID) COVTYPE(DIAG)

/EMMEANS=TABLES(OVERALL).

>******************************Overall trust Correlations with gaze ratio of looking at traffic signal during crossing*********************************************

MIXED ZTrustForDrivingBehavior WITH ZCrossRatio_traffic

/CRITERIA=CIN(95) MXITER(100) MXSTEP(10) SCORING(1) SINGULAR(0.000000000001) HCONVERGE(0,

ABSOLUTE) LCONVERGE(0, ABSOLUTE) PCONVERGE(0.000001, ABSOLUTE)

/FIXED=ZCrossRatio_traffic | SSTYPE(3)

/METHOD=REML

/PRINT=CORB COVB G SOLUTION TESTCOV

/RANDOM=INTERCEPT | SUBJECT(SubjectID) COVTYPE(VC)

/REPEATED=ScenarioID | SUBJECT(SubjectID) COVTYPE(DIAG)

/EMMEANS=TABLES(OVERALL).

>******************************Overall trust Correlations with gaze ratio of looking at task elements during crossing*********************************************

MIXED ZTrustForDrivingBehavior WITH ZCrossRatio_task

/CRITERIA=CIN(95) MXITER(100) MXSTEP(10) SCORING(1) SINGULAR(0.000000000001) HCONVERGE(0,

ABSOLUTE) LCONVERGE(0, ABSOLUTE) PCONVERGE(0.000001, ABSOLUTE)

/FIXED=ZCrossRatio_task | SSTYPE(3)

/METHOD=REML

/PRINT=CORB COVB G SOLUTION TESTCOV

/RANDOM=INTERCEPT | SUBJECT(SubjectID) COVTYPE(VC)

/REPEATED=ScenarioID | SUBJECT(SubjectID) COVTYPE(DIAG)

/EMMEANS=TABLES(OVERALL).

>******************************Overall trust Correlations with gaze ratio of looking at pedestrian signals during crossing*********************************************

MIXED ZTrustForDrivingBehavior WITH ZCrossRatio_Signal

/CRITERIA=CIN(95) MXITER(100) MXSTEP(10) SCORING(1) SINGULAR(0.000000000001) HCONVERGE(0,

ABSOLUTE) LCONVERGE(0, ABSOLUTE) PCONVERGE(0.000001, ABSOLUTE)

/FIXED=ZCrossRatio_Signal | SSTYPE(3)

/METHOD=REML

/PRINT=CORB COVB G SOLUTION TESTCOV

/RANDOM=INTERCEPT | SUBJECT(SubjectID) COVTYPE(VC)

/REPEATED=ScenarioID | SUBJECT(SubjectID) COVTYPE(DIAG)

/EMMEANS=TABLES(OVERALL).

>******************************Overall trust Correlations with gaze ratio of looking at other areas in the environment during crossing*********************************************

MIXED ZTrustForDrivingBehavior WITH ZCrossRatio_untagged

/CRITERIA=CIN(95) MXITER(100) MXSTEP(10) SCORING(1) SINGULAR(0.000000000001) HCONVERGE(0,

ABSOLUTE) LCONVERGE(0, ABSOLUTE) PCONVERGE(0.000001, ABSOLUTE)

/FIXED=ZCrossRatio_untagged | SSTYPE(3)

/METHOD=REML

/PRINT=CORB COVB G SOLUTION TESTCOV

/RANDOM=INTERCEPT | SUBJECT(SubjectID) COVTYPE(VC)

/REPEATED=ScenarioID | SUBJECT(SubjectID) COVTYPE(DIAG)

/EMMEANS=TABLES(OVERALL).

>********************************************************Gaze correlatins during Task********************************************************************

>******************************Overall trust Correlations with gaze ratio of looking at vehicle during task*********************************************

MIXED ZTrustForDrivingBehavior WITH ZTaskRatio_look_vehicle

/CRITERIA=CIN(95) MXITER(100) MXSTEP(10) SCORING(1) SINGULAR(0.000000000001) HCONVERGE(0,

ABSOLUTE) LCONVERGE(0, ABSOLUTE) PCONVERGE(0.000001, ABSOLUTE)

/FIXED=ZTaskRatio_look_vehicle | SSTYPE(3)

/METHOD=REML

/PRINT=CORB COVB G SOLUTION TESTCOV

/RANDOM=INTERCEPT | SUBJECT(SubjectID) COVTYPE(VC)

/REPEATED=ScenarioID | SUBJECT(SubjectID) COVTYPE(DIAG)

/EMMEANS=TABLES(OVERALL).

>******************************Overall trust Correlations with gaze ratio of checking for vehicle during task*********************************************

MIXED ZTrustForDrivingBehavior WITH ZTaskRatio_check_vehicle

/CRITERIA=CIN(95) MXITER(100) MXSTEP(10) SCORING(1) SINGULAR(0.000000000001) HCONVERGE(0,

ABSOLUTE) LCONVERGE(0, ABSOLUTE) PCONVERGE(0.000001, ABSOLUTE)

/FIXED=ZTaskRatio_check_vehicle | SSTYPE(3)

/METHOD=REML

/PRINT=CORB COVB G SOLUTION TESTCOV

/RANDOM=INTERCEPT | SUBJECT(SubjectID) COVTYPE(VC)

/REPEATED=ScenarioID | SUBJECT(SubjectID) COVTYPE(DIAG)

/EMMEANS=TABLES(OVERALL).

>******************************Overall trust Correlations with gaze ratio of looking at crosswalk during task*********************************************

MIXED ZTrustForDrivingBehavior WITH ZTaskRatio_crosswalk

/CRITERIA=CIN(95) MXITER(100) MXSTEP(10) SCORING(1) SINGULAR(0.000000000001) HCONVERGE(0,

ABSOLUTE) LCONVERGE(0, ABSOLUTE) PCONVERGE(0.000001, ABSOLUTE)

/FIXED=ZTaskRatio_crosswalk | SSTYPE(3)

/METHOD=REML

/PRINT=CORB COVB G SOLUTION TESTCOV

/RANDOM=INTERCEPT | SUBJECT(SubjectID) COVTYPE(VC)

/REPEATED=ScenarioID | SUBJECT(SubjectID) COVTYPE(DIAG)

/EMMEANS=TABLES(OVERALL).

>******************************Overall trust Correlations with gaze ratio of looking at traffic signal during task*********************************************

MIXED ZTrustForDrivingBehavior WITH ZTaskRatio_traffic

/CRITERIA=CIN(95) MXITER(100) MXSTEP(10) SCORING(1) SINGULAR(0.000000000001) HCONVERGE(0,

ABSOLUTE) LCONVERGE(0, ABSOLUTE) PCONVERGE(0.000001, ABSOLUTE)

/FIXED=ZTaskRatio_traffic | SSTYPE(3)

/METHOD=REML

/PRINT=CORB COVB G SOLUTION TESTCOV

/RANDOM=INTERCEPT | SUBJECT(SubjectID) COVTYPE(VC)

/REPEATED=ScenarioID | SUBJECT(SubjectID) COVTYPE(DIAG)

/EMMEANS=TABLES(OVERALL).

>******************************Overall trust Correlations with gaze ratio of looking at task elements during task*********************************************

MIXED ZTrustForDrivingBehavior WITH ZTaskRatio_task

/CRITERIA=CIN(95) MXITER(100) MXSTEP(10) SCORING(1) SINGULAR(0.000000000001) HCONVERGE(0,

ABSOLUTE) LCONVERGE(0, ABSOLUTE) PCONVERGE(0.000001, ABSOLUTE)

/FIXED=ZTaskRatio_task | SSTYPE(3)

/METHOD=REML

/PRINT=CORB COVB G SOLUTION TESTCOV

/RANDOM=INTERCEPT | SUBJECT(SubjectID) COVTYPE(VC)

/REPEATED=ScenarioID | SUBJECT(SubjectID) COVTYPE(DIAG)

/EMMEANS=TABLES(OVERALL).

>******************************Overall trust Correlations with gaze ratio of looking at pedestrian signal during task*********************************************

MIXED ZTrustForDrivingBehavior WITH ZTaskRatio_Signal

/CRITERIA=CIN(95) MXITER(100) MXSTEP(10) SCORING(1) SINGULAR(0.000000000001) HCONVERGE(0,

ABSOLUTE) LCONVERGE(0, ABSOLUTE) PCONVERGE(0.000001, ABSOLUTE)

/FIXED=ZTaskRatio_Signal | SSTYPE(3)

/METHOD=REML

/PRINT=CORB COVB G SOLUTION TESTCOV

/RANDOM=INTERCEPT | SUBJECT(SubjectID) COVTYPE(VC)

/REPEATED=ScenarioID | SUBJECT(SubjectID) COVTYPE(DIAG)

/EMMEANS=TABLES(OVERALL).

>******************************Overall trust Correlations with gaze ratio of looking at other areas in the environment during task*********************************************

MIXED ZTrustForDrivingBehavior WITH ZTaskRatio_untagged

/CRITERIA=CIN(95) MXITER(100) MXSTEP(10) SCORING(1) SINGULAR(0.000000000001) HCONVERGE(0,

ABSOLUTE) LCONVERGE(0, ABSOLUTE) PCONVERGE(0.000001, ABSOLUTE)

/FIXED=ZTaskRatio_untagged | SSTYPE(3)

/METHOD=REML

/PRINT=CORB COVB G SOLUTION TESTCOV

/RANDOM=INTERCEPT | SUBJECT(SubjectID) COVTYPE(VC)

/REPEATED=ScenarioID | SUBJECT(SubjectID) COVTYPE(DIAG)

/EMMEANS=TABLES(OVERALL).

>**********************************************************************************************************************************************************************************

>**********************************************************************************************************************************************************************************
